# Variants in Doublecortin- and Calmodulin Kinase Like 1, a Gene Up-Regulated by BDNF, Are Associated with Memory and General Cognitive Abilities

**DOI:** 10.1371/journal.pone.0007534

**Published:** 2009-10-21

**Authors:** Stéphanie Le Hellard, Bjarte Håvik, Thomas Espeseth, Harald Breilid, Roger Løvlie, Michelle Luciano, Alan J. Gow, Sarah E. Harris, John M. Starr, Karin Wibrand, Astri J. Lundervold, David J. Porteous, Clive R. Bramham, Ian J. Deary, Ivar Reinvang, Vidar M. Steen

**Affiliations:** 1 Bergen Mental Health Research Center, Department of Clinical Medicine, University of Bergen, Bergen, Norway; 2 Dr Einar Martens' Research Group for Biological Psychiatry, Center for Medical Genetics and Molecular Medicine, Haukeland University Hospital, Helse Bergen HF, Bergen, Norway; 3 Center for the Study of Human Cognition, Department of Psychology, University of Oslo, Blindern, Oslo, Norway; 4 Centre for Cognitive Ageing and Cognitive Epidemiology, Department of Psychology, University of Edinburgh, Edinburgh, United Kingdom; 5 Centre for Cognitive Ageing and Cognitive Epidemiology, Medical Genetics Section, University of Edinburgh Centre for Molecular Medicine, Western General Hospital, Edinburgh, United Kingdom; 6 Department of Biomedicine and Bergen Mental Health Research Center, University of Bergen, Bergen, Norway; 7 Department of Biological and Medical Psychology, University of Bergen, Bergen, Norway; James Cook University, Australia

## Abstract

**Background:**

Human memory and general cognitive abilities are complex functions of high heritability and wide variability in the population. The brain-derived neurotrophic factor (BDNF) plays an important role in mammalian memory formation.

**Methodology / Principal Finding:**

Based on the identification of genes markedly up-regulated during BDNF-induced synaptic consolidation in the hippocampus, we selected genetic variants that were tested in three independent samples, from Norway and Scotland, of adult individuals examined for cognitive abilities. In all samples, we show that markers in the doublecortin- and calmodulin kinase like 1 (*DCLK1*) gene, are significantly associated with general cognition (IQ scores) and verbal memory function, resisting multiple testing. *DCLK1* is a complex gene with multiple transcripts which vary in expression and function. We show that the short variants are all up-regulated after BDNF treatment in the rat hippocampus, and that they are expressed in the adult human brain (mostly in cortices and hippocampus). We demonstrate that several of the associated variants are located in potential alternative promoter- and cis-regulatory elements of the gene and that they affect BDNF-mediated expression of short *DCLK1* transcripts in a reporter system.

**Conclusion:**

These data present *DCLK1* as a functionally pertinent gene involved in human memory and cognitive functions.

## Introduction

Empirical evidence for genetic factors underlying cognitive variation is strong, but identification of specific genetic variants has proved challenging. The study of differences in human mental abilities, measured with psychometric tools, has defined a hierarchical structure of human cognition. General cognitive ability (*g*) stands at the pinnacle, accounting for about 50% of the total variance in test performances. Correlated with *g*, there are additional, separable group factors representing distinct cognitive domains, with memory as a prominent example [1], [2]. Behavioural studies suggest that the impact of genetic factors on specific cognitive domains, e.g. memory, derives from both inherited contributions to general cognitive ability *and* from genetic variation that more selectively influences memory. This heritability is thought to be polygenic but, to date, few molecular genetic studies have provided replicated genetic associations with cognitive function [3].

Variations in the gene for the brain-derived neurotrophic factor (*BDNF*) have been examined for associations between cognitive traits and psychiatric disorders. Several studies have identified association of a single nucleotide polymorphism (SNP - rs6265) – which corresponds to an amino acid change (Val66Met) – and verbal memory, general cognitive ability, age related change in reasoning skills and hippocampal functions, but others have failed to replicate these findings [4], [5], [6], [7], [8], [9]. The same Val66Met genetic variant of BDNF has been largely studied as a risk factor for bipolar affective disorder, schizophrenia and other psychiatric disorders (for review see [10], [11]). Since several of the cognitive traits associated with BDNF variants have been reported potentially deficient in major psychosis (e.g. verbal memory and general cognition in bipolar disorder and/or schizophrenia [12], [13]), these common associations might reflect genetic associations to clinical endophenotypes of these disorders. Indeed, neurocognitive traits have been proposed as endophenotypes for psychosis that, because they are less clinically heterogeneous and have high heritability, would be more powerful in identifying genetic factors of susceptibility [14], [15], [16].

Functional convergent genomics approaches, which use a set of candidate genes identified in gene expression-based relevant models [17], [18], [19], have been successful to identify new genes for schizophrenia, antipsychotic induced weight gain and bipolar disorders [20], [21], [22]. In order to broaden the study of *BDNF* in cognitive traits, we chose to develop a functional convergent genomics approach to characterise genes up-regulated by BDNF, and their implication in cognition. BDNF plays a critical role as a trigger of memory formation and transcription-dependent enhancement of synaptic strength [23], via neuronal activity induced gene expression [24]. Previously, we used a rat model where BDNF is infused in the dentate gyrus region of the hippocampal formation, and identified a panel of 14 genes that were up-regulated following BDNF treatment that induces long-term potentiation – LTP, in a similar way as the immediate early gene *Arc* [25]. Among these genes, the five most strongly differentially expressed genes were further examined by *in situ* hybridization and also found to be up-regulated in another LTP inducing model (afferent high frequency stimulation of the synaptic strength in the dentate gyrus). In the present study, we selected haplotype-tagging SNPs to screen those genes (*ARL4L*, *NEURITIN, DCLK1*, *KLF10* and *NPTX2*) together with *BDNF* and *ARC* for influence on human cognitive functioning in samples of healthy individuals who volunteered for testing of their memory and general intellectual function (IQ).

## Materials and Methods

### 1. Samples descriptions

All Norwegian participants read an information sheet and signed a statement of informed consent approved by the regional ethical committee for medical research (Project ID: S-03116). Permission to obtain and store blood samples for genotyping together with cognitive and MRI data in a biobank was given by the Department of Health, and permission to establish a registry with relevant information for a time period of 10 years was given by the Department of Health. For the Scottish samples, ethics permission for the study protocol was obtained from the Multi-Centre Research Ethics Committee for Scotland (MREC/01/0/56) and from Lothian Research Ethics Committee (LREC/2003/2/29). The research was carried out in compliance to the Helsinki Declaration. All subjects gave written, informed consent.

**The Norwegian Cognitive NeuroGenetics (NCNG) sample.** consists of 271 participants aged 46–75 years (mean age of 62.6 years, s.d. 7.9) that were recruited through advertisements in local newspapers in the Oslo and Bergen urban areas (see previous report in Espeseth et al., 2006 [26]). Candidates were first interviewed by phone according to a check list about health and previous illness or injuries. Exclusion criteria were previously diagnosed neurological or psychiatric illness, any other chronic illness that might influence test performance, or sensory or motor impairments. Participants with a history of alcohol or substance abuse, or current addictive disorders, were also excluded. Participants had to be native speakers of Norwegian and have completed obligatory basic education (7 years for this age group) without diagnosed reading or learning disorders. Persons on adequate medication for hypertension, diabetes or hypercholesterolemia were not excluded. Participants were not allowed to consume nicotine or caffeine during the test period or in the lab premises, but were not required to abstain from these substances prior to attendance. There were 195 females (72%) and the average total years of education was 13.9 (s.d. 3.0). The Vocabulary and Matrix Reasoning subscales of the Wechsler Abbreviated Scale of Intelligence (WASI, [27]) were used to estimate IQ, and the California Verbal Learning Test II (CVLT-II, [28]) to test verbal episodic memory.

**The Lothian Birth Cohort 1921 (LBC1921).** are surviving participants of the Scottish Mental Survey of 1932 (SMS1932). They were recruited in Edinburgh and the surrounding areas either through the Community Health Index – a list of individuals registered with a General Practitioner – or as volunteers replying to media calls [29]. The first wave of follow-up ran from 1999–2001, and 550 individuals (234 men and 316 women) were tested individually at the Wellcome Trust Clinical Research Facility (WTCRF) at the Western General Hospital, Edinburgh. The mean age of the LBC1921 participants was 10.9 years (s.d. 0.3) at the time of the SMS1932 and 79.1 years (s.d. 0.6) at wave 1 assessment.

In the SMS1932, all participants completed the Moray House Test (MHT) Number 12 which was re-administered at age 79. The Moray House Test is a well-validated IQ-type test with a predominance of verbal reasoning items, though there are also some other types of item. The raw MHT scores were corrected for age in days at the time of testing and then converted into IQ scores. At wave 1, a further battery of cognitive tests was also completed, including the Raven's Progressive Matrices to assess non-verbal reasoning [30], Verbal Fluency to assess executive function [31], and Logical Memory to assess verbal declarative memory [32]. The Mini-Mental State Examination as a brief screen for dementia [33] and the Hospital Anxiety and Depression Scale [34] were also administered. Further description can be found in Deary et al. (2004) [29].

In the present study, only the association with IQ score, logical memory (immediate, delayed and total), verbal fluency and Raven's matrices were analyzed as these variables were the most comparable to the variables analyzed in the NCNG sample.

**The Lothian Birth Cohort 1936 (LBC1936).** comprises 1091 participants who were born in 1936 and tested on a general measure of verbal reasoning (Moray House Test No. 12; MHT) at age 11 in the Scottish Mental Survey of 1947 (Scottish Council for Research in Education [SCRE], [35]). They were recruited in Edinburgh and the surrounding areas either through the Community Health Index —a list of individuals registered with a General Practitioner— or as volunteers replying to media calls [36]. All participants lived independently in the community and were able to travel to the clinical research facility for testing. They undertook medical and cognitive testing at age 70 (mean age of 69.6 years, s.d. 0.8) as reported in detail previously [36], including the same MHT test as they had taken at age 11. The raw MHT scores were corrected for age in days at the time of testing and then converted into IQ scores. The Mini-Mental State Examination (MMSE) was used to screen for possible dementia [33]. The battery of cognitive tests sampled a variety of specific cognitive abilities, with an emphasis on memory and processing speed. Memory domains were assessed by the following subtests of the Wechsler Memory Scale-IIIUK (WMS-IIIUK; [37]): Logical Memory I (immediate verbal declarative memory), Logical Memory II (delayed verbal declarative memory), Verbal paired associates (immediate and delayed verbal memory and learning) and Spatial span (non-verbal, spatial memory). The information processing speed battery comprised two psychometric tests from the WAIS-IIIUK (Digit symbol coding and Symbol search) and two elementary cognitive tasks, Reaction Time (simple and choice conditions) and Inspection Time. Other cognitive tests which tapped diverse abilities included: Backward digit span (working memory) from the Wechsler Memory Scale-IIIUK; and Letter-number sequencing (working memory), Matrix reasoning (non-verbal reasoning) and Block design (constructional ability) from the WAIS-IIIUK [37]. The Verbal fluency test provided a measure of executive function [31].The *g* factor was calculated via principal components analysis of the following Wechsler tests: Backward digit span, Letter-number sequencing, Matrix reasoning, Block design, Digit Symbol and Symbol Search subtests. A full description of these tests can be found in the Lothian Birth Cohort 1936 protocol article [36]. The final sample for analysis (i.e., those who also had genotype data) was N  =  1077, and included 535 females and 542 males.

### 2. Selection, genotyping and analysis of genetic markers

#### Markers selection, genotyping and analysis in the NCNG sample

Haplotype tagging markers (single nucleotide polymorphisms – SNP) were selected using the Phase I (16c.1, June05, based on NCBI B34 assembly, dbSNP b124) version of HapMap (http://www.hapmap.org/cgi-perl/gbrowse/gbrowse/hapmap_phaseI/).[38], and according to the protocol described in Christoforou et al. [39]. Briefly, Hapmap data for the CEU trios were downloaded and analysed in Haploview v2.5, using the following criteria: pair-wise comparisons of markers more than 500 kb apart were ignored, minor allele frequency ≥0.10, a Hardy–Weinberg (HW) *P*-value ≥0.001, genotyping success rate ≥0.75. The haplotype blocks were defined using the solid spine of LD approach, using Haploview's internal tagging program. Haplotype tagging SNPs were selected on a block-by-block basis to represent haplotypes of frequencies higher than or equal to 0.10. The markers were genotyped on a Sequenom Massarray platformTM (http://www.sequenom.com/, Sequenom Inc., San Diego, CA, USA) at CIGENE, Center for Integrative Genetics (Universitetet for miljø- og biovitenskap, Ås, Norway, http://www.umb.no/), which is the national FUGE platform for genotyping (www.fuge.no), supported by the Research Council of Norway.

#### Markers selection, genotyping and analysis in the LBC1921 and LBC1936 samples

In the replication sample we decided to concentrate only on the *DCLK1* gene. Seven markers that showed association in the NCNG sample (p-value <0.01: rs4591003, rs1926467, rs943220, rs10507435, rs7323560, rs7334245, rs9315383) were selected for replication in the LBC samples. In addition, we chose to include another eight markers for genotyping in these Scottish samples, since they show association to psychiatric disorders in preliminary studies (unpublished information): rs12430800, rs2296645, rs10492555, rs872060, rs9545332, rs7989245, rs7989807 and rs9315390. The marker rs4391923 was also included for its potential functional relevance.

Assays were developed for the ABI PRISM® 7900HT Sequence Detection System using TaqMan technology (Applied Biosystems, CA, USA), with genotyping at the Welcome Trust Clinical Research Facility, Genetics Core (http://www.wtcrf.ed.ac.uk/genetics/default%20genetics.htm, University of Edinburgh, UK).

### Data analysis

Our discovery sample (the NCNG, N = 271) provides 93%, 73% and 30% power to detect an additive QTL effect explaining 5%, 3% and 1% of the trait variance, respectively (uncorrected P = 0.05; two-tailed), calculated with the Genetic Power Calculator, http://pngu.mgh.harvard.edu/~purcell/gpc/ [40].

Genotypes were quality controlled with the following criteria: individual samples with genotype call rate <90% and markers with call rate <96% or Hardy Weinberg P-value <0.001 were excluded from analysis (see supplementary material –SOM, for summary of locations, markers tested and analysed).

Genotyping data were analyzed using the Helix Tree software for linear regression, genotypic association and haplotype trend regression of 2- and 3- markers sliding windows, similarly to the analysis described in [20].

#### CNV considerations

Copy Number Variants (CNVs) were not considered at the time of the markers selection (few data were available at this time, September 2005). Retrospectively, the Toronto database (http://projects.tcag.ca/variation/) was screened for CNVs in the genes investigated. Markers in the *NPTX2*, *BDNF* and *DCLK1* genes are not affected by any known CNVs. *ARC* is located within the rare variation_30296 seen in 1 in 1086 chromosomes by Jakobsson et al. [41], *KLF10* is located within the CNV region of variation_7659 described by de Smith et al. [42] observed in 1 in 100 chromosomes, and *NRN1* is located on the same clone as the variation_0072 seen in 1 in 110 chromosomes by Iarfate et al. [43] which has not been further refined. Considering their relatively low frequency, and the Hardy Weinberg equilibrium observed for genetic variants of this region in our genotyping, we consider that none of these known CNVs are likely to affect the associations reported in this study.

### 3. RT-PCR of human *DCLK1* transcripts

cDNA samples were synthesized from human brain region-specific total RNA samples (Clontech Laboratories, CA, USA) (200 ng RNA input) using the SuperScript III First-Strand Synthesis System (Life Technologies, St Paul, MN, USA), according to manufacturer's protocol (20 µl reaction volume). 0,5 µl cDNA was used as template in 10 µl real-time PCR assays (50°C/2 min; 95°C/10 min; 40×(95°C/15 sec; 60°C/1 min)). Real-time PCR analyses were performed with an ABI Prism 7900^HT^ sequence detector system (Applied Biosystems, CA, USA) using SYBR-green (Eurogentec, Belgium) as detector, AmpliTaq Gold DNA polymerase (Applied Biosystems, CA, USA), 2x SYBR-green mix, and the following PCR primer sets:

*CARP*: 5′-GGATGACTTGGATTCAGTAGGAGACT, 5′-CATGGTTAGTGTGTTCTTGTACTCAATATT;

long *DCLK1*: 5′-GGAGTGGTGAAACGCCTGTAC, 5′- GGTTCCATTAACTGAGCTGG;

short *DCLK1*: 5′-ACACTAAGACTGTGTCCATGTTAGAACTC, 5′-AAGCCTTCCTCCGACACTTCT.

The specificity of all RT-PCR assays was verified by DNA-sequencing of the amplified PCR products.

### 4. Semi-quantitative RT-PCR of BDNF-induced expression of *Dclk1 in vivo*

The expression of *Dclk1* transcripts in the rat hippocampus were measured by semi-quantitative RT-PCR on cDNA samples obtained from our previous study on BDNF-mediated induction of Long-Term Potentiation in the rat hippocampus [25]. PCR were performed as described above with the following primer sets: *Dclk1* long: 5′-GGTGTGGTGAAGCGTCTGTAC, 5′-CAAAAAAGTCCTGAAGGCACATC;*Dclk1* short: 5′-ACACTAAGACTGTGTCCATGTTAGAACTC, 5-GGCCATCGTTCTCATCCATT. The primer sets for *Carp* and *Neuritin* has been described elsewhere [25].

### 5. Sequencing of intron 5 of human DCLK1

Genomic DNAs of 23 individuals selected for their genotypes at the markers rs943220 and rs10507435 from the NCNG sample were amplified to sequence the DCLK1 intron 5. Nineteen pairs of primers for PCR amplification (sequences can be made available upon request) of the intron 5 were designed, using the Primer3 tool [44] (http://fokker.wi.mit.edu/primer3/input.htm). The PCR amplicons were designed to have a sufficient (100–150 bp) overlap to obtain contiguous sequences. PCR fragments were amplified using AmpliTaq Gold (Applied Biosystems, Foster City, CA, USA) according to the manufacturer's instructions with 25 cycles: 94°C for 10 s, 55°C for 30 s, 72°C for 30 s (initial denaturation 94°C for 10 min). The PCR products were sequenced with BigDye v3.1 (Applied Biosystems) according to the manufacturer's instructions and the sequences were aligned with phredPhrap program and read in Consed39 [45] (http://bozeman.mbt.washington.edu/consed/consed.html#documentation).

All new polymorphism identified have been deposited in dbSNP (http://www.ncbi.nlm.nih.gov/sites/entrez?db=snp, build 130) under the following IDs: rs61949282, rs61949292, rs66492553, rs67014603, rs72652874, rs72652875, rs72652876, rs72652877, rs72652878, rs72652879, rs72652880, rs72652881, rs72652882, rs72652883, rs72652884

### 6. *In silico* prediction of promoter and cis-regulatory regions in intron 5 of human *DCLK1*

Candidate gene-regulatory regions were identified on the basis of clustering of transcription factor (TF) DNA-binding sites, as identified by the TF-search engine (www.cbrc.jp/research/db/TFSEARCH) and the Cis-element Cluster Finder (Cister)-program (http://zlab.bu.edu/%7Emfrith/cister.shtml) using matrixes from TRANSFAC (http://www.gene-regulation.com). TFs predicted to bind proximal to SNPs verified by sequencing in the human *DCLK1* intron 5 were selected for the Cister-analyses.

The Neuronal Network Promoter Prediction program was used to rank candidate TATA-boxes in intron 5 (http://www.fruitfly.org/seq_tools/promoter.html). Probability scores of clustering between promoter elements and TATA-boxes were investigated by the Cister-program and the TF-search engine.

### 7. Construction, transfections and assays of the Luciferase reporter vectors

#### Construction of luciferase reporter vectors

The predicted promoter fragments were amplified from genomic DNA (from 2 individuals homozygous for the C or T allele of rs4391923/marker m5.3) by PCR with AmpliTaq Gold DNA polymerase (Applied Biosystems), using the forward primer 5′-CTAGACTCGAGCCTCCTGAAGATAGCTTTGC and the reverse primer 5′- CAGACAAGCTTCAGTCTCAGGAATACCTTGC (XhoI and HindIII sites introduced, underlined). Purified PCR fragments were cloned into the XhoI/HindIII (all restriction enzymes from New England Biolabs, Ipswich, MA, USA) sites of pGL4.11[luc2P] (Promega, Madison, WI, USA), generating the pGL4.11-Cprom and pGL4.11-Tprom luciferase reporters.

Three haplotypes of the intron 5 cis2 element (hap1a, hap1b and hap2, see section 6 and supplementary online material - SOM) were PCR amplified from genomic DNA (from 3 individuals homozygous for the three haplotypes identified by sequencing), using the forward primer 5′-GTCACGGATCCTTGGAAACTCAAGAAGATAGGC and the reverse primer 5′- GATCAGTCGACCCACAGGAAACAAAGCAACC (BamHI and Sal1 sites introduced, underlined). Amplified DNA was cloned into the BamH1/Sal1 sites of pGL4.11-Cprom, pGL4.11-Tprom and pGL4.11[luc2P], generating haplotype specific cis2-C/Tprom luciferase reporters and promoter-less control vectors. In the final plasmids, the Cis2 element locates 2216 bp upstream of the promoter-region, the two being separated by a synthetic poly(A) sequence for the reduction of background signals. All plasmid constructions were verified by DNA sequencing. See supplementary material –SOM, for a list of commercial and constructed plasmids used in the study.

#### Cell cultures, differentiation and plasmid transfection

SH-SY5Y cells (ATCC, LGC Promochem, UK) were grown in RPMI supplemented with 10% (v/v) horse fetal serum, 5% (v/v) fetal bovine serum and 2 mM L-glutamine (Cambrex Biosciences, Cambrex Corporation, Charles City, IA, USA). Cultures were incubated at 37°C in a 5 vol-% CO_2_/air incubator.

Plasmid transfections were performed over-night using MetafecteneTM Pro (Biontex, Munchen, Germany) under conditions optimized according to the manufacturer's guidelines. Neuronal differentiation was initiated the day after transfection by replacing the transfection media with fresh media containing BDNF (50 ng/ml)/RA (10 µM all-trans retinoic acid) (Sigma-Aldrich, St Louis, MO, USA), as described by Holback et al. [46]. Control cells (non-differentiated) were treated similarly but exposed to media with vehicle only (0.01% DMSO). Cell viability was monitored using WST-1 (F. Hoffmann-La Roche Ltd, Germany). All transfections were performed in quadruplicates on 96 multi-well plates. Each experiment was controlled for transfection efficiency by transfecting a green fluorescent protein (GFP) expressing vector (pSIREN-RetroQ-ZsGreen) into a separate set of cells. GFP expression was analyzed two days post transfection on a FACSCalibur™ Flow Cytometer (BD systems, BD Europe, Belgium).

#### Luciferase Reporter Assays

The transcriptional activity of each reporter plasmid was measured two days post transfection using the Dual-Luciferase Reporter Assay system (Promega Biotech AB, Madison, WI, USA), according to manufacturer's protocol. Luciferase signal intensities were recorded on a Chameleon plate reader (Reactionlab Sverige AB, Sweden). All plasmid transfections and luciferase measurements were performed in 96 multiwell plates, with quadruplicates of each sample. Each experiment was repeated 5 times for the pGL4-C/Tprom plasmids, and 7 times for plasmids harboring cis2 elements. All experiments were performed blind to the vectors haplotypes. Luciferase signal intensities were normalized for transfection efficiency on the basis of renilla luciferase signal intensities from the co-transfected pGL4-73[hRluc/SV40] plasmid. The transcriptional activity of a given reporter plasmid is listed relative to its respective promoter-less control vector. All cis2 promoter-less control vectors showed similar background activity (data not shown).

## Results

### Association to verbal memory and general cognition in the NCNG sample

In the original study design, seven genes were selected: *BDNF*,*ARC*, *ARL4L*, *NEURITIN, DCLK1*, *KLF10* and *NPTX2*. During quality check of the genotyping results, we noticed that the official annotation of the rat *Arl4l* had been updated to *Arf4l* and that the human homolog we selected (*ARL4L*) was erroneous and should have been *ARL4D*. We therefore decided to exclude the *ARL4L* markers from further analysis as this gene is apparently not up-regulated during LTP in rat brain.

We excluded DNA samples with a genotyping success below 0.9, and markers with call rate <96% or Hardy Weinberg P-value <0.001 from analysis. For the 6 remaining genes studied, a total of 48 markers were genotyped in the Norwegian Cognitive NeuroGenetics —NCNG —sample. This sample consists of 271 individuals (mean age: 62.6 years, range 50–75), which had been recruited via media advertisements and who were subjected to cognitive testing, i.e. verbal memory and general cognition (IQ score). In this sample, we found strong associations between several markers in the *DCLK1* gene (doublecortin- and calmodulin kinase like 1, a.k.a. *DCAMKL1*) and aspects of verbal memory function and IQ score, which resisted Bonferroni correction (see Table 1, Table 2 and SOM for all results of markers tested). The most significant associations were observed for intron 5 markers m5.1 (rs10507435) and m5.2 (rs943220), where the less frequent genotypes were significantly associated with reduced verbal memory performance (see Table 1). Marker m5.1 was also associated with IQ score, like several other *DCLK1* SNPs (see Table 2).

**Table 1:** Genotypic single marker association analysis to verbal memory traits.

					NCNG		LBC1921	LBC1936
					(N = 271)	(n = 550)	(N = 1077)
	Markers	CVLTIICVLTII	LM	LM
Gene	tested	associated	code	localization	Learning	Delayed recall	Delayed	Learning
***NRN1***	7	-	-	-	-	-	n.t	n.t
***NPTX2***	3	-	-	-	-	-	n.t	n.t
***KLF10***	4	-	-	-	-	-	n.t	n.t
***ARC***	2	-	-	-	-	-	n.t	n.t
***BDNF***	6	-	-	-	-	-	n.t	n.t
***DCLK1***	26	rs9315383	m3.1	intron 3	0.0033	-	-	-
		rs7334245	m3.2	intron 3	0.0059	-	-	-
		rs7989245	m4	intron 4	-	-	-	-
		rs10507435	m5.1	intron 5	0.0021	**0.00043**	-	-
		rs943220	m5.2	intron 5	0.0036	**0.00010**	-	-
		rs4391923	m5.3	intron 5	-	-	-	0.0067
		rs2296645	m11	exon 11			0.0068	

Analyses were performed with Helix Tree software. Only p-values <0.01 are reported (see SOM for regression analysis, marker details, results and description of cognitive traits tested). In the NCNG sample, verbal learning and delayed recall were assessed with the California Verbal Learning Test (CVLT-II [28]). In total, six BDNF-LTP related genes were tested and 48 markers were analyzed. In the LBC samples, verbal memory was tested with the Wechsler memory scale test (LM- delayed and learning [32], [37]) and the samples were genotyped for 16 *DCLK1* markers only, with no screening of the other genes (n.t: not tested). P-values below Bonferroni corrected p-value threshold ( = 0.001 for NCNG), are highlighted in bold.

**Table 2:** Association of *DCLK1* markers to IQ scores in the three samples.

			NCNG	LBC1921	LBC1936
			IQ	IQ79	IQ79rIQ11	IQ11	IQ70
Markers	Code	Localisation	LR	HTR3	LR	HTR3	LR	HTR3	LR	HTR3	LR	HTR3
rs10492555	m5′.1	5′	-	0.034	-	-	-	-	-	-	-	-
rs9315390	m5′.2	5′	-	0.010	-	-	-	-	-	-	-	-
rs9315383	m3.1	Intron 3	0.005	-	-	-	-	-	-	-	-	-
rs7334245	m3.2	Intron 3	-	-	-	-	-	-	-	-	-	-
rs7989807	m3.3	Intron 3	-	-	-	-	-	-	-	-	-	-
rs7323560	m3.4	Intron 3	-	-	-	-	-	-	-	-	-	-
rs7989245	m4	Intron 4	-	-	-	-	-	-	-	-	-	-
rs10507435	m5.1	Intron 5	0.027	-	-	-	-	-	-	-	-	**0.0092**
rs943220	m5.2	Intron 5	-	-	-	-	-	-	-	-	-	-
rs4391923	m5.3	Intron 5	-	-	-	-	-	-	-		0.010	-
rs2296645	m11	Exon 11	-	0.046	-	-	-	-	-	0.045		-
rs1926467	m15	Intron 15	0.025	-	-	-	0.014	-	0.023	-	-	-
rs12430800	m19.1	Intron 19	-	0.019	**0.0042**	0.022	**0.0019**	**0.00034**	-	-	-	-
rs4591003	m19.2	Intron 19	-	-	-	0.014	0.039	-	0.015	-	-	-
rs9545332	m19.3	Intron 19	-	-	-		-	-	0.023	-	-	-
rs872060	m3′	3′	-	-	0.026	-	0.038	-	-	-	-	-

Both single marker linear regression (LR) and haplotype trend regression of 3-markers sliding windows analyses (HTR3) are presented. Only p-values below 0.05 are displayed. All analyses were performed using sex and age as covariates. The NCNG was assessed with the Wechsler Abbreviated Scale of Intelligence [27]. LBC1921 and LBC1936 were assessed for IQ with the Moray House Test (see SOM). For LBC1921, no association below p = 0.05 was observed for IQ11. In the LBC samples, all regression analyses were performed both with and without IQ11 as a covariate. P-values highlighted in bold are resisting a 10,000 permutations testing.

For the other genes tested we found some significant effects, but no strong association (p<0.01) or associations across several cognitive traits (see SOM). However, in a 2-loci analysis for gene-gene interaction effects, there were significant interactions between *DCLK1* intron 5 markers (m5.1 and m5.2) and markers tagging *BDNF* and *ARC* on the association with verbal memory (p-value  = 0.03−0.003, after Bonferroni correction, see SOM). At the genetic level, this finding is consistent with the co-upregulation of *Dclk1* transcription by Arc and Bdnf, as observed in a rat hippocampal model of synapse consolidation.

### Replication in the Lothian Birth Cohorts

Sixteen *DCLK1* SNPs were selected for replication testing in two independent samples (see SOM). The Scottish Lothian Birth Cohort (LBC) studies of individuals born in 1921 and in 1936 (LBC1921 and LBC1936) are two independent cohorts of individuals who underwent an IQ test at age 11 (IQ11), and who at the age of 79 years for the LBC1921, and 70 years for the LBC1936, participated in follow-up testing with examination of IQ (IQ79 and IQ70, respectively) and other cognitive functions [29], [36].

In the LBC1921, we found an association between verbal memory and m11, an exon 11 synonymous marker (see Table 1). For IQ79, especially when regressed for IQ11, we replicated and further strengthened the association to markers in the intron 15 and intron 19 (which are located in the same haplotype block), and to intron 19 3-markers haplotype (resisting permutation testing, see Table 2).

In the LBC1936, we observed associations between an intron 5 marker (m5.3) and several memory-specific and general cognition traits at age 70 (see Table 1, and 2). Also in this sample, the association with intron 5 was even stronger (resisting permutation testing), for both memory and general cognition traits, at the haplotype level (3-markers haplotype covering the intron 5, see Table 2). In this sample, the intron 15 – intron 19 markers (except for m19.1, rs12430800) were associated with childhood cognitive variables (IQ11). Further association testing with other cognitive abilities (see SOM) showed an effect of markers in both the intron 5 and intron 15–19, but also an effect of the age at testing. This age-dependent association is especially pronounced for the general cognitive ability factor, *g* factor, which was associated with both intron 5 (m5.3, rs4391923) and intron 15 - intron 19, but only the intron 5 association remained significant when IQ11 was adjusted for in the regression model.

### Expression of rat *Dclk1* variants after BDNF treatment in the dentate gyrus

**Figure 1:**
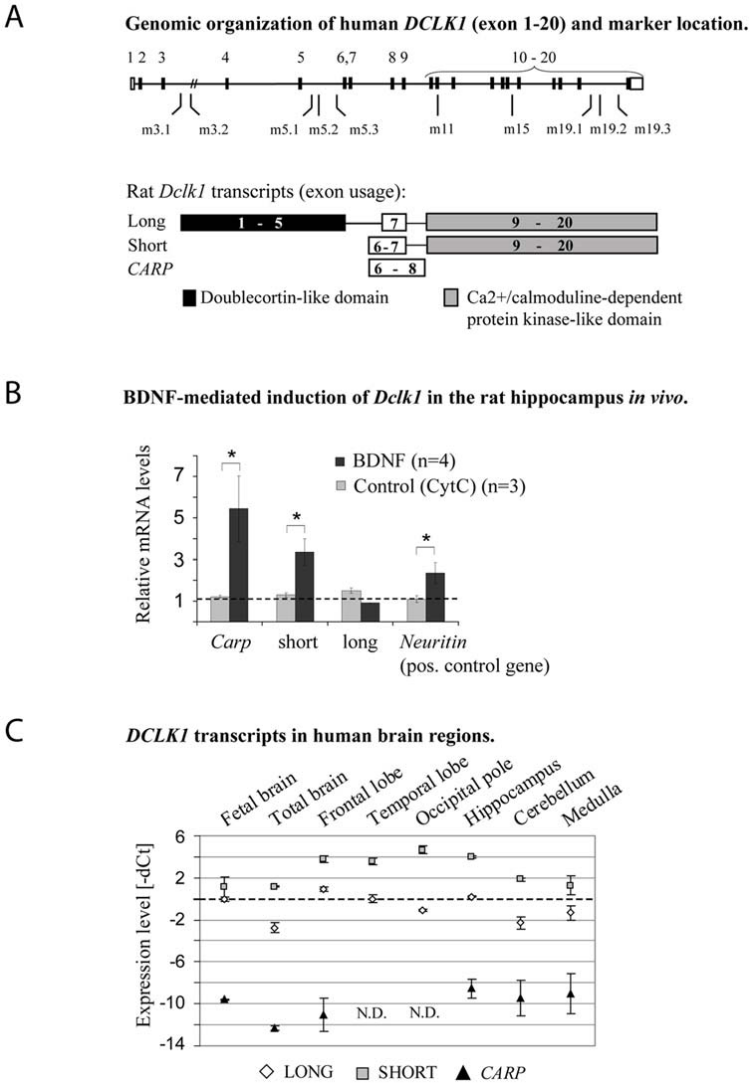
Genomic organization of human *DCLK1* and expression of transcript variants in human brain regions and in rat hippocampus in response to BDNF. (A) Proposed genomic organization, exon usage and marker location for the human *DCLK1* gene. In rodents, *Dclk1* contains 20 exons producing several transcripts, such as long, short and *Carp* mRNAs. In human, the reference sequence only lists 18 exons encoding the long *DCLK1* transcript (NM_004734). With transcript-specific RT-PCR assays (see below, panel C), we show that short *DCLK1* and *CARP* are expressed in humans (with inclusion of exon 6 and 8), thus the human genomic sequence should contain 20 exons. Black, white and grey boxes illustrate protein domains encoded by different exons. The genomic locations of markers with positive scores or interactions are marked. (B) The expression of *Dclk1* variants in response to infusion of exogenous BDNF into the dentate gyrus *in vivo* were analyzed by real-time RT PCR. BDNF mediates the expression of short *Dclk1* variants and *Carp*, while the long (full-length) *Dclk1* transcripts are unaffected (or slightly reduced). Infusion of Cytochrome C was used as a negative control. (C) RT-PCR amplification of N-terminal- (“long”) and C-terminal (“short”) domain *DCLK1* transcripts and *CARP* expressed in human brain regions. –dCt values are given relative to the Ct of long *DCLK1* in fetal brain (dotted line, Ct = 22.9, mean ± S.E.M.). N.D.: not detected / variable detection.

The rodent *Dclk1* gene is expressed as several transcripts, e.g., *long* (exons 1–20 except 6 and 8), *short* (exons 6–20 except 8) and *Carp* (exons 6–8; see figure 1A), which vary in expression (spatial and developmental) and in function (see discussion). In rat, we previously demonstrated that the *Carp* variant is induced by BDNF exposure, but at this time we did not further look at the other transcripts. Further examination of the samples from that study, show that all short *DCLK1* variants are induced by BDNF but not the long variants (see figure 1B).

### Expression of *DCLK1* variants in human brain tissues

In the rodent, the short variants show higher expression in adult brains whereas long variants dominate during embryonic stages in rat [47]. In humans, the expression of *DCLK1* variants has not been fully documented, but expression of long *DCLK1* has been shown in both embryonic and adult brain tissues [48]. Using transcript-specific PCR assays, we found expression of both long and short transcripts of *DCLK1* in the fetal brain (26–40 weeks) as well as in specific regions of the adult human brain (see Figure 1C). The expression of short variants was especially high in regions involved in memory performance (hippocampus, occipital pole, frontal lobe and temporal lobe). The expression of *CARP* was low in all human brain tissues, consistent with previous observations in rodents of low basal *CARP* expression and robust induction by specific treatments [25], [49].

### *In silico* characterisation of regulatory elements and identification of additional genetic variants in the intron 5 of *DCLK1*

**Figure 2:**
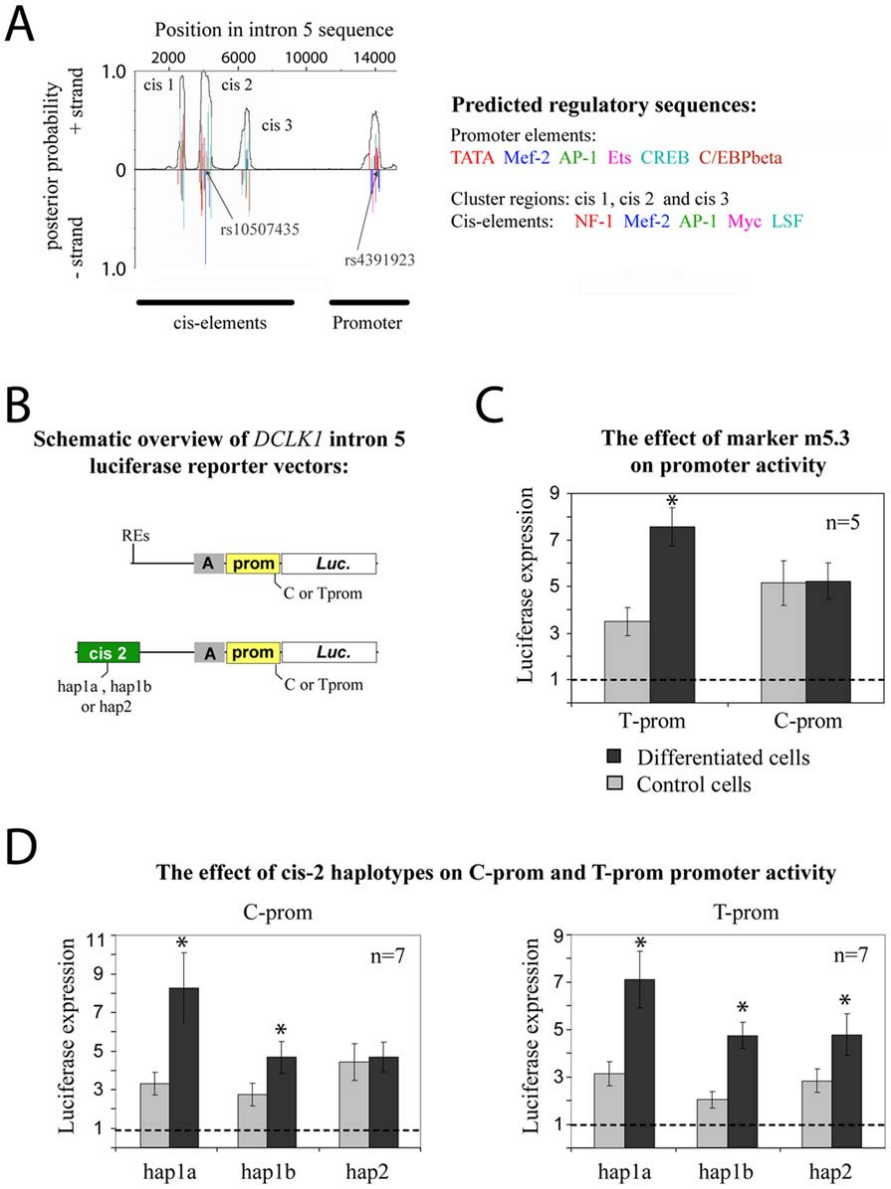
*In silico* prediction and functional characterization of promoter elements in intron 5 of human *DCLK1*. (A) Illustration of probability scores for transcription factor binding-sites in three clusters and a promoter proximal region (merged pictures, see SOM). Arrows indicate the position of SNP markers. See SOM for cis1-3 and promoter chromosomal coordinates. (B) Schematic representation of luciferase reporter vectors. Abbreviations: Prom, promoter; luc, luciferase reporter gene. (C) Luciferase reporter assay demonstrating basal promoter activity and allele-specific responses to neuronal differentiation of SH-SY5Y cells. (D) Luciferase reporter assays demonstrating cis2 haplotype-specific effects in response to neuronal differentiation of SH-SY5Y cells. Luciferase signal intensities obtained from non-differentiated cells (grey bars) and RA/BDNF-exposed cells (black bars) are compared for each reporter plasmid. Values are given as mean ± S.E.M. * Statistically significant different expression in differentiated cells as compared to control cells (t-test, p<0.05).

Considering that one of the main signals of association was observed for markers in the intron 5 of *DCLK1* and that short transcripts of *DCLK1* all start from the exon 6, we hypothesised that the intron 5 might harbour alternative promoter and regulatory regions. We searched for transcriptional cis-regulatory elements and promoters in the human intron 5 with the aim of analyzing the effect of these in luciferase reporter assays. The search predicted a TATA-box promoter and three specific cis-regulatory elements (cis1-3, see Figure 2A). To identify additional sequence variants that could affect the regulatory elements, we sequenced the entire intron 5 from genomic DNA of 23 individuals from the NCNG sample. In the putative alternative promoter region in intron 5, the marker m5.3 (C/T variant) was located 2 bp from the potential transcription start. For the adjacent regulatory elements we observed several variants in the cis2 that could affect the regulatory properties, defining three haplotypes: the related hap1a and hap1b, and the hap2 (see SOM for details).

### Assessment of the functional effect of SNPs in potential regulatory regions on the expression of *DCLK1* short variants

Luciferase reporter assays were constructed by cloning the promoter C (C-prom) or T allele (T-prom) of m5.3, from genomic DNAs of NCNG individuals. Additional reporter assays were constructed to mimic *in vitro* the potential effect of the 3 cis2 haplotypes on the promoter. The assays were analysed for expression of the reporter luciferase protein under control conditions and under BDNF/RA differentiation (retinoic acid, protocol for BDNF induced differentiation as described by Holback et al. [46]).

In luciferase reporter assays, both the C-prom and T-prom constructs displayed basic promoter reporter-activity in the undifferentiated SH-SY5Y control cells. However, during BDNF-induced neuronal differentiation, only the T-prom construct demonstrated increased expression of the luciferase reporter (see Figure 2C). The addition of a single hap1a or hap1b variant of the cis2 element into the non-inducible C-prom reporter-vector rendered the C-prom inducible by BDNF (see Figure 2D), whereas the hap2 variant of cis2 did not show this effect on C-prom. None of the cis2 variants had any significant additional effect on the activity and inducibility of the T-prom sequence. These reporter assays thus demonstrated that the predicted promoter in intron 5 displays an allele-specific promoter activity and inducibility which can be further influenced by regulatory elements, such as cis2, in an allele-specific mode.

## Discussion

Our data show that genetic variants in *DCLK1* significantly influence the performance on tests of memory and intellectual function in three independent samples. Although the size of our discovery sample is small, we detected association in this sample. We observed further associations between genetic variants in *DCLK1* and cognitive abilities in the replication samples but with differences regarding the panel of markers that were associated. These differences could be due to type I errors, but also to type II errors by lack of power for either the discovery or the replication samples to detect association. However it is probable that these differences are due to allelic heterogeneity that reflects the variation between the samples. Even though all subjects have been phenotyped for similar traits (e.g. verbal memory and general cognition), they are different in the geographical origin, age, or mode of recruitment, as well as in the specific tests used to assess cognitive phenotypes.

Comparison of the associations in the three samples highlights three main regions of interest in the *DCLK1* gene that could have an influence on the heritability of cognitive traits. The first region extends from the 5′UTR to the intron 3 of the gene, where the markers m3.1 and m3.2 in single marker allelic analysis or certain 3-markers haplotypes in haplotype trend regression are associated to verbal memory or to general cognition in the NCNG sample (see Tables 1 and 2), to IQ at age 79 in the LBC1921 (see SOM) and to several cognitive traits at the genotypic level in the LBC1936. The region spanned by these markers is large and covers the promoter region and the first exons and introns. Notably, it includes a large proportion of the wide intron 3, which contains a large number of highly conserved non-coding elements as seen in the UCSC browser (http://genome.ucsc.edu/cgi-bin/hgGateway).

The second region of association is localised in intron 5 of the gene. This area is associated to verbal memory and IQ at the single marker level in the NCNG sample (see Table 1 and 2), to verbal memory and several other cognitive traits at the single marker level (m5.3, see Table 1 and SOM) and to IQ at age 70 at the 3-markers haplotype level in the LBC1936 (see Table 2 and SOM). Several short transcripts for the *DCLK1* start from the exon 6 (as further detailed below). We characterised a potential promoter in intron 5, which may be used for transcription of the short variants, as well as three regions with potential regulatory effect on this transcription. Our reporter assay studies of this putative promoter and one of the regulatory regions (chosen because it encompasses associated genetic variants) displayed that the efficiency of these regulatory elements was influenced by the alleles of the associated markers and by the treatment with BDNF. In this context, it is interesting to note that the BDNF- inducible C-prom/cis2 haplotypes (hap 1a/b), equivalent to 2-marker m5.1-m5.3_GA haplotype of intron 5, shows the strongest association to IQ score and verbal memory (p-value  = 0.0027, see SOM and 0.0019, data not shown) in the LBC1936, and that the “non-inducible” constructs corresponds to the m5.1-m5.3 GG haplotype that is the rarest, found only in 2.5% of the chromosomes.

The third region of association points to a haplotype block of markers covering intron 15 to intron 19. This region is associated to IQ score in the NCNG sample at the single marker and 3-markers haplotype level (see Table 2), to IQ79 in the LBC1921 (single marker and three markers haplotype, see Table 2) and to IQ11 (see Table 2) and to other cognitive variables (see SOM) at the single marker level in the LBC1936. This association calls for additional studies, but it is interesting to note that the associated markers are located near exon 19 that is alternatively spliced in several transcripts, affecting the DCLK1 kinase activity [50], within a region of high inter-species conservation (as seen in the UCSC: http://genome.ucsc.edu/cgi-bin/hgGateway).

In addition, in both the LBC1921 and LBC1936, single marker association to the marker rs2296645 were observed for verbal memory and cognitive abilities. This genetic variant is located in the exon 11 but does not affect the amino acid sequence.

The thorough examination of the Lothian Birth Cohorts, especially the LBC1936, shows that the signal of association depends on the cognitive variable studied and on the age at examination, which points to similarities and differences in genetic contributions to cognitive abilities across the lifespan. Several cognitive associations in old age were affected by the integration of childhood IQ as a covariate, which can suggest that these are genetic associations to lifelong cognitive change.

In addition, we show that there might be interaction between variants in *DCLK1* and variants in *BDNF* and *ARC* for an effect on verbal memory and general cognition. This finding is coherent at the genetic level with the *in vivo* observation that *Dclk1* is co-upregulated with *Arc* by BDNF in the rat hippocampus [25]. In addition to the known implication of BDNF in long term potentiation, the interactions with Arc are also noteworthy as this immediate early gene is required for multiple forms of synaptic plasticity and long-term memory formation, including synaptic potentiation induced by BDNF infusion [51], [52], [53].

Considering the conflicting results reported regarding the genetic association of *BDNF* with human cognition and with psychiatric disorders [5], [6], [7], [8], [9], [10], we suggest that re-analysis of the possible *BDNF-DCLK1* interaction might improve the interpretation of these studies. In this perspective, it is also interesting to notice that in two recent studies of functional convergent genomics for bipolar affective disorder [22] and a genome wide scan for personality traits [54], both *BDNF* and *DCLK1* (a.k.a. *DCAMKL1* in these reports) were ranked as strong candidates.

The rodent *Dclk1* gene is expressed as several transcripts, e.g., *long* (exons 1–20 except 6 and 8), *short* (exons 6–20 except 8) and *Carp* (exons 6–8; see figure 1A), which vary in expression (spatial and developmental) and in function. The long transcripts encode an N-terminal domain similar to the lissencephalia-related doublecortin gene, sharing its microtubule-binding and -stabilizing properties [55], [56]. N-terminal *Dclk1* transgenic and *Dcl* knockdown mice develop brain abnormalities that affect the organization of hippocampal neurons, cortical neurogenesis, neuronal migration and axonal wiring [56], [57]. The C-terminal domain, present in both long- and short-*Dclk1*, contains a domain similar to the Ca2+/calmodulin-dependent protein kinase which may phosphorylate the myelin basic protein [47], [50], [58]. The function of Carp remains largely unknown but it is expressed in response to diverse stimuli, and may be involved in both neuronal activity-induced strengthening of synaptic transmission as well as apoptosis [59]. We now show that the short variants are, similarly to *Carp*, up-regulated by BDNF treatment in the rat hippocampus, and that in human these variants are expressed in brain structures relevant to cognition.

In conclusion, in this study we report *DCLK1*, a gene up-regulated BDNF, as being a novel candidate gene associated to cognitive traits, in three different samples, but with allelic heterogeneity between the samples. Further work will be needed to better understand the function(s) of DCLK1 in cognitive processes, and its role in synaptic plasticity, especially since other related genes (*CAMK2G* and *CAMTA1*) have also been associated to human memory performance [60], [61]. Drawing on the example of this study, it will be interesting to mine ongoing genome wide studies of large samples characterised for cognitive abilities with further gene sets identified from micro-array gene expression experiments on models relevant to synaptic plasticity.

### Supplementary information – SOM

with additional material and methods and complementary tables and figures, is linked to the online version of the paper (see Supporting Information Material S1).

## Supporting Information

Supporting Information Material S1 Methods and Tables. Revised version of the supplementary information, as accepted after resubmission 1.
(1.52 MB DOC)
