# Supplementary material for: Variants in Doublecortin- and Calmodulin Kinase Like 1, a Gene Up-Regulated by BDNF, Are Associated with Memory and General Cognitive Abilities
Source: PLoS One. 2009 Oct 21;4(10):e7534. doi: 10.1371/journal.pone.0007534 (PMC2760101; doi:10.1371/journal.pone.0007534)
Supplement: Supporting Information Material S1 — Methods and Tables. Revised version of the supplementary information, as accepted after resubmission 1. (1.52 MB DOC) [file pone.0007534.s001.doc]

Supplementary Online Material – SOM: **Variants in doublecortin- and calmodulin kinase like 1, a gene up-regulated by BDNF, are associated with memory and general cognitive abilities.**

Stéphanie Le Hellard^1,2^,*, Bjarte Håvik^1,2^,*, Thomas Espeseth^3^, Harald Breilid^1,2^, Roger Løvlie^1,2^, Michelle Luciano^4^, Alan J. Gow^4^, Sarah E. Harris^5^, John M. Starr^4^, Karin Wibrand^6^, Astri J. Lundervold^7^, David J. Porteous^5^, Clive R. Bramham^6^, Ian J. Deary^4‡^, Ivar Reinvang^3‡^ and Vidar M. Steen^1,2^

1. Bergen Mental Health Research Center, Department of Clinical Medicine, University of Bergen, N-5021 Bergen, Norway.
2. Dr Einar Martens’ Research Group for Biological Psychiatry, Center for Medical Genetics and Molecular Medicine, Haukeland University Hospital, Helse Bergen HF, N-5021 Bergen, Norway.
3. Center for the Study of Human Cognition, Department of Psychology, University of Oslo, Box 1094, Blindern, N-0317 Oslo, Norway.
4. Centre for Cognitive Ageing and Cognitive Epidemiology, Department of Psychology, University of Edinburgh, EH8 9JZ Edinburgh, UK.
5. Centre for Cognitive Ageing and Cognitive Epidemiology, Medical Genetics Section, University of Edinburgh Centre for Molecular Medicine, Western General Hospital, EH4 2XU Edinburgh, UK.
6. Department of Biomedicine and Bergen Mental Health Research Center, University of Bergen, Jonas Lies vei 91, 5009 Bergen, Norway.
7. Department of Biological and Medical Psychology, University of Bergen, Jonas Lies vei 91, 5009 Bergen, Norway.

*^,‡^ these authors contributed equally to this study.

Corresponding author:

Stephanie le Hellard,

Center for Medical Genetics and Molecular Medicine, Haukeland University Hospital, Helse Bergen HF, N-5021 Bergen, Norway.

E-mail: [stephanie.hellard@med.uib.no](mailto:stephanie.hellard@med.uib.no)

Tel: (+47) 55 97 53 37

Fax: (+47) 55 97 54 79.

Keywords: DCAMKL1, CARP, LTP, IQ, transcriptional regulatory elements.

[I. SUPPLEMENTARY ONLINE MATERIAL AND METHODS 3](#__RefHeading___Toc224461205)

[a) Markers summary 3](#__RefHeading___Toc224461206)

[b) List of commercial and constructed plasmids used in the study: 4](#__RefHeading___Toc224461207)

[II. Supplementary Online Tables and Figures 5](#__RefHeading___Toc224461208)

[A. Data from all markers results in the NCNG sample. 5](#__RefHeading___Toc224461209)

[1. SOM Table 1: marker statistics in the NCNG sample. 5](#__RefHeading___Toc224461210)

[2. SOM Table 2. Single marker genotypic analysis in the NCNG sample. 6](#__RefHeading___Toc224461211)

[3. SOM Table 3. Single marker regression analysis in the NCNG sample. 7](#__RefHeading___Toc224461212)

[4. SOM table 4: Haplotype trend regression in the NCNG sample. 9](#__RefHeading___Toc224461213)

[5. SOM table 5: HTR with LBC markers in the NCNG sample. 10](#__RefHeading___Toc224461214)

[6. SOM Table 6: Two genetic loci interaction analyses for the CVLT II-30 min delayed recall trait, in the NCNG sample. 10](#__RefHeading___Toc224461215)

[B. Data from all DCLK1 markers typed in the LBC1921 11](#__RefHeading___Toc224461216)

[1. SOM Table 7: Markers statistics in the LBC1921 11](#__RefHeading___Toc224461217)

[2. SOM Table 8: Genotypic analysis in the LBC1921. 11](#__RefHeading___Toc224461218)

[3. SOM Table 9: Linear regression analysis in the LBC1921. 12](#__RefHeading___Toc224461219)

[4. SOM Table 10: Haplotype trend regression analysis in the LBC1921 13](#__RefHeading___Toc224461220)

[C. Data from all DCLK1 markers typed in the LBC1936 13](#__RefHeading___Toc224461221)

[1. SOM Table 11: Marker statistics in the LBC1936. 13](#__RefHeading___Toc224461222)

[2. SOM Table 12: Genotypic analysis in the LBC1936. 14](#__RefHeading___Toc224461223)

[3. SOM Table 13: Linear regression analysis in the LBC1936. 17](#__RefHeading___Toc224461224)

[4. SOM Table 14: Haplotype trend regression analysis in the LBC1936 19](#__RefHeading___Toc224461225)

[D. Results from sequencing of human DCLK1 intron 5. 19](#__RefHeading___Toc224461226)

[E. Prediction of promoter and regulatory regions of human DCLK1 intron 5. 21](#__RefHeading___Toc224461227)

[F. Expression of human CARP mRNA in cultured SH-SY5Y cells. 22](#__RefHeading___Toc224461228)

[III. References 22](#__RefHeading___Toc224461229)

# SUPPLEMENTARY ONLINE

# MATERIAL AND METHODS

#### Markers summary

The following table summarizes for each gene 1) the chromosome location 2) the coordinates used for download, 3) the most centromeric and telomeric markers from the download, 4) the number of htSNPs selected, 5) the number of assay that failed on design and could not be replaced by another SNP in linkage disequilibrium, 6) the number of assays that failed at the genotyping (genotyping success <0.96, or SNP monomorphic), 7) the number of SNPs which had a Hardy Weinberg Disequilibrium (p-value < 0.001), and 8) the number of SNPs analysed.

| **Gene** | **1** | **2** | **3** | **4** | **5** | **6** | **7** | **8** |
| --- | --- | --- | --- | --- | --- | --- | --- | --- |
| *ARC* | 8q24.3 | chr8:143,581,286-143,603,531 | rs9324593-rs10097505 | 3 | 1 |  |  | 2 |
| *BDNF* | 11p14.1 | chr11:27,630,751-27,717,605 | rs925946-rs1491851 | 7 | 1 |  |  | 6 |
| *DCLK1* | 13q13.3 | chr13:34,133,478-34,513,443 | rs872060-rs1410641 | 32 | 1 | 4 | 1 | 26 |
| *KLF10* | 8q22.3 | chr8:103,607,604-103,634,535 | rs2033563-rs2511730 | 5 |  | 1 |  | 4 |
| *NPTX2* | 7q22.1 | chr7:97,848,006-97,880,577 | rs2697490-rs6465693 | 4 |  | 1 |  | 3 |
| *NRN1* | 6p25.1 | chr6:5,933,234-5,962,632 | rs11758973-rs605865 | 8 |  | 1 |  | 7 |

#### List of commercial and constructed plasmids used in the study:

*Name: Description:*

pGL4.11[luc2P] Promoter-less luciferase reporter plasmid.

pGL4.11-Cpro Promoter luciferase reporter plasmid, with C allele of rs4391923.

pGL4.11-Tpro Promoter luciferase reporter plasmid, with T allele of rs4391923.

pGL4.11-hap1a Promoter-less cis2 control plasmid, with cis 2 hap1a haplotype.

pGL4.11-hap1b Promoter-less cis2 control plasmid, with cis 2 hap1b haplotype.

pGL4.11-hap2 Promoter-less cis2 control plasmid, with cis 2 hap2 haplotype.

pGL4.11-hap1a-Cprom Cis2-promoter luciferase reporter plasmid, with C allele of rs4391923 and cis 2 hap1a haplotype.

pGL4.11-hap1b-Cprom Cis2-promoter luciferase reporter plasmid, with C allele of rs4391923 and cis 2 hap1b haplotype.

pGL4.11-hap2-Cprom Cis2-promoter luciferase reporter plasmid, with C allele of rs4391923 and cis 2 hap2 haplotype.

pGL4.11-hap1a-Tprom Cis2-promoter luciferase reporter plasmid, with T allele of rs4391923 and cis 2 hap1a haplotype.

pGL4.11-hap1b-Tprom Cis2-promoter luciferase reporter plasmid, with T allele of rs4391923 and cis 2 hap1b haplotype.

pGL4.11-hap2-Tprom Cis2-promoter luciferase reporter plasmid, with T allele of rs4391923 and cis 2 hap2 haplotype.

pSIREN-RetroQ-ZsGreen Constitutive expression of GFP (Clontech, Palo Alto, CA, USA)

pGL4-73[hRluc/SV40] Constitutive expression of renilla luciferase (Promega)

# Supplementary Online Tables and Figures

## Data from all markers results in the NCNG sample.

### SOM Table 1: marker statistics in the NCNG sample.

**SNPids** are according to dbSNP ( <http://www.ncbi.nlm.nih.gov/projects/SNP/> ) reference sequence entries. **Chr** and **position**: chromosomal position of the SNP according to NCBI36 build, with the name of the **gene** that the SNP (**D/d**) is tagging; **MAF**: Minor Allele Frequency; **CR:** Call Rate, i.e. Sequenom genotyping success rate; **HWE P**: Hardy Weinberg Equilibrium p-value. Genotypes (**DD, Dd, dd and failed**) or Allele (**D, d and failed**) are given in counts.

| **SNP id** | **chr** | **position** | **Gene** | **D** | **d** | **MAF** | **CR** | **HWE P** | **DD** | **Dd** | **dd** | **Failed** | **D** | **d** | **Failed** |
| --- | --- | --- | --- | --- | --- | --- | --- | --- | --- | --- | --- | --- | --- | --- | --- |
| **rs2038640** | **6** | **5,933,747** | **NRN1** | C | T | 0.3408 | 0.99 | 0.28 | 35 | 112 | 120 | 4 | 182 | 352 | 8 |
| **rs17363382** | **6** | **5,935,822** | **NRN1** | G | A | 0.0759 | 1.00 | 0.21 | 3 | 35 | 232 | 1 | 41 | 499 | 2 |
| **rs10484319** | **6** | **5,938,995** | **NRN1** | C | G | 0.3064 | 0.98 | 0.78 | 24 | 115 | 127 | 5 | 163 | 369 | 10 |
| **rs12333117** | **6** | **5,939,990** | **NRN1** | C | T | 0.4349 | 0.99 | 0.64 | 49 | 136 | 84 | 2 | 234 | 304 | 4 |
| **rs582186** | **6** | **5,946,379** | **NRN1** | A | G | 0.3481 | 1.00 | 0.38 | 36 | 116 | 118 | 1 | 188 | 352 | 2 |
| **rs3763180** | **6** | **5,954,846** | **NRN1** | G | T | 0.4513 | 0.99 | 0.69 | 56 | 129 | 82 | 4 | 241 | 293 | 8 |
| **rs594734** | **6** | **5,962,211** | **NRN1** | A | T | 0.2195 | 0.97 | 0.01 | 20 | 75 | 167 | 9 | 115 | 409 | 18 |
| **rs11971348** | **7** | **98,090,205** | **NPTX2** | C | G | 0.1493 | 0.99 | 0.99 | 6 | 68 | 194 | 3 | 80 | 456 | 6 |
| **rs1728987** | **7** | **98,098,162** | **NPTX2** | G | C | 0.3922 | 0.99 | 0.26 | 37 | 137 | 95 | 2 | 211 | 327 | 4 |
| **rs6465693** | **7** | **98,104,773** | **NPTX2** | A | G | 0.0593 | 1.00 | 0.95 | 1 | 30 | 239 | 1 | 32 | 508 | 2 |
| **rs1434278** | **8** | **103,724,969** | **KLF10** | A | T | 0.2119 | 0.99 | 0.98 | 12 | 90 | 167 | 2 | 114 | 424 | 4 |
| **rs2436969** | **8** | **103,726,284** | **KLF10** | A | G | 0.1444 | 1.00 | 0.86 | 6 | 66 | 198 | 1 | 78 | 462 | 2 |
| **rs980112** | **8** | **103,734,688** | **KLF10** | T | C | 0.0936 | 0.99 | 0.81 | 2 | 46 | 219 | 4 | 50 | 484 | 8 |
| **rs2511730** | **8** | **103,739,213** | **KLF10** | C | G | 0.1181 | 1.00 | 0.20 | 6 | 52 | 213 | 0 | 64 | 478 | 0 |
| **rs11167152** | **8** | **143,688,078** | **Arc** | C | G | 0.431 | 0.99 | 0.96 | 50 | 131 | 87 | 3 | 231 | 305 | 6 |
| **rs10097505** | **8** | **143,691,185** | **Arc** | A | G | 0.4793 | 0.98 | 0.83 | 62 | 131 | 73 | 5 | 255 | 277 | 10 |
| **rs1519479** | **11** | **27,624,106** | **BDNF** | C | T | 0.5 | 0.99 | 0.85 | 68 | 133 | 68 | 2 | 269 | 269 | 4 |
| **rs6265** | **11** | **27,636,491** | **BDNF** | A | G | 0.1845 | 1.00 | 0.19 | 6 | 88 | 177 | 0 | 100 | 442 | 0 |
| **rs12273363** | **11** | **27,701,434** | **BDNF** | C | T | 0.2148 | 1.00 | 0.60 | 11 | 94 | 165 | 1 | 116 | 424 | 2 |
| **rs908867** | **11** | **27,702,339** | **BDNF** | A | G | 0.0762 | 0.99 | 0.21 | 3 | 35 | 231 | 2 | 41 | 497 | 4 |
| **rs1491850** | **11** | **27,706,300** | **BDNF** | C | T | 0.4387 | 0.99 | 0.85 | 51 | 134 | 84 | 2 | 236 | 302 | 4 |
| **rs1491851** | **11** | **27,709,338** | **BDNF** | T | C | 0.4627 | 0.99 | 0.06 | 65 | 118 | 85 | 3 | 248 | 288 | 6 |
| **rs10492555** | **13** | **35,607,108** | **DCLK1** | A | C | 0.1468 | 0.99 | 0.28 | 8 | 63 | 198 | 2 | 79 | 459 | 4 |
| **rs9315390** | **13** | **35,606,999** | **DCLK1** | T | C | 0.2103 | 1.00 | 0.46 | 14 | 86 | 171 | 0 | 114 | 428 | 0 |
| **rs1001232** | **13** | **35,606,207** | **DCLK1** | G | T | 0.1157 | 0.99 | 0.15 | 6 | 50 | 212 | 3 | 62 | 474 | 6 |
| **rs1926324** | **13** | **35,582,442** | **DCLK1** | C | T | 0.2368 | 0.98 | 0.51 | 13 | 100 | 153 | 5 | 126 | 406 | 10 |
| **rs6563330** | **13** | **35,557,362** | **DCLK1** | C | G | 0.2462 | 0.97 | 1.00 | 16 | 98 | 150 | 7 | 130 | 398 | 14 |
| **rs9315383** | **13** | **35,549,854** | **DCLK1** | C | G | 0.4815 | 1.00 | 0.70 | 61 | 138 | 71 | 1 | 260 | 280 | 2 |
| **rs7334245** | **13** | **35,545,152** | **DCLK1** | C | T | 0.4004 | 0.98 | 0.24 | 38 | 137 | 91 | 5 | 213 | 319 | 10 |
| **rs1926317** | **13** | **35,542,039** | **DCLK1** | A | G | 0.4114 | 1.00 | 0.33 | 42 | 139 | 90 | 0 | 223 | 319 | 0 |
| **rs7989807** | **13** | **35,523,088** | **DCLK1** | C | T | 0.1404 | 0.99 | 0.89 | 5 | 65 | 197 | 4 | 75 | 459 | 8 |
| **rs7323560** | **13** | **35,510,464** | **DCLK1** | C | A | 0.0498 | 1.00 | 0.67 | 1 | 25 | 245 | 0 | 27 | 515 | 0 |
| **rs8000458** | **13** | **35,419,986** | **DCLK1** | T | C | 0.0722 | 1.00 | 0.59 | 2 | 35 | 233 | 1 | 39 | 501 | 2 |
| **rs7989245** | **13** | **35,397,596** | **DCLK1** | C | T | 0.3026 | 1.00 | 0.73 | 26 | 112 | 133 | 0 | 164 | 378 | 0 |
| **rs1926452** | **13** | **35,342,936** | **DCLK1** | A | G | 0.1362 | 0.99 | 0.99 | 5 | 63 | 200 | 3 | 73 | 463 | 6 |
| **rs10507435** | **13** | **35,338,995** | **DCLK1** | G | A | 0.2156 | 0.99 | 0.37 | 15 | 86 | 168 | 2 | 116 | 422 | 4 |
| **rs943220** | **13** | **35,337,251** | **DCLK1** | C | T | 0.3609 | 0.98 | 0.72 | 36 | 120 | 110 | 5 | 192 | 340 | 10 |
| **rs2148417** | **13** | **35,331,861** | **DCLK1** | A | G | 0.0387 | 1.00 | 0.51 | 0 | 21 | 250 | 0 | 21 | 521 | 0 |
| **rs4391923** | **13** | **35,328,855** | **DCLK1** | A | G | 0.2344 | 0.94 | 0.47 | 12 | 96 | 148 | 15 | 120 | 392 | 30 |
| **rs1926466** | **13** | **35,326,594** | **DCLK1** | C | T | 0.4404 | 0.96 | 0.16 | 56 | 117 | 87 | 11 | 229 | 291 | 22 |
| **rs9574707** | **13** | **35,301,834** | **DCLK1** | A | G | 0.1648 | 1.00 | 0.46 | 9 | 71 | 190 | 1 | 89 | 451 | 2 |
| **rs2296645** | **13** | **35,300,425** | **DCLK1** | A | G | 0.476 | 1.00 | 0.41 | 58 | 142 | 71 | 0 | 258 | 284 | 0 |
| **rs1926467** | **13** | **35,291,374** | **DCLK1** | A | G | 0.4963 | 1.00 | 0.90 | 67 | 134 | 69 | 1 | 268 | 272 | 2 |
| **rs7328001** | **13** | **35,271,238** | **DCLK1** | A | G | 0.0852 | 1.00 | 0.97 | 2 | 42 | 226 | 1 | 46 | 494 | 2 |
| **rs12430800** | **13** | **35,257,973** | **DCLK1** | C | T | 0.2132 | 0.98 | 0.99 | 12 | 89 | 164 | 6 | 113 | 417 | 12 |
| **rs4591003** | **13** | **35,256,049** | **DCLK1** | C | T | 0.4593 | 1.00 | 0.63 | 55 | 138 | 77 | 1 | 248 | 292 | 2 |
| **rs9545332** | **13** | **35,253,679** | **DCLK1** | C | T | 0.1384 | 1.00 | 0.54 | 4 | 67 | 200 | 0 | 75 | 467 | 0 |
| **rs872060** | **13** | **35,236,916** | **DCLK1** | C | T | 0.1125 | 1.00 | 0.79 | 3 | 55 | 213 | 0 | 61 | 481 | 0 |

### SOM Table 2. Single marker genotypic analysis in the NCNG sample.

The markers were analysed for genotypic association to the following variables:

- California Verbal Learning Test (**CVLT-II**) performances for the **Learning**, **5 min free recall** and **30 minutes free recall** parameters. *In the main text delayed recall refers to 30 minutes free recall.*
- Wechsler Abbreviated Scale of Intelligence test (**WASI-IQ**).
- Number of **Years of Education** is highly correlated to scores of verbal memory and general cognition traits and is therefore presented as additional information only and was not taken into consideration for selection of markers for subsequent studies.

The genotypic analyses (performed with Helix Tree) compare with an F-test the mean value of the variable for the genotype considered, binned in a two- versus one genotype comparison.

| **SNP id** | **chr** | **position** | **Gene** | **CVLT Learning** | **CVLT 5 min free recall** | **CVLT 30 min free recall** | **WASI IQ** | **Years of education** |
| --- | --- | --- | --- | --- | --- | --- | --- | --- |
| **rs2038640** | **6** | **5,933,747** | **NRN1** | 0.1210 | 0.1231 | **0.0834** | 0.1054 | 0.3914 |
| **rs17363382** | **6** | **5,935,822** | **NRN1** | 0.2773 | 0.5258 | 0.5833 | 0.4236 | **0.0393** |
| **rs10484319** | **6** | **5,938,995** | **NRN1** | 0.7419 | 0.6497 | 0.5235 | 0.3037 | **0.0331** |
| **rs12333117** | **6** | **5,939,990** | **NRN1** | 0.2490 | 0.2605 | 0.1898 | 0.6555 | 0.4422 |
| **rs582186** | **6** | **5,946,379** | **NRN1** | 0.4104 | 0.5953 | 0.6906 | 0.7157 | **0.0895** |
| **rs3763180** | **6** | **5,954,846** | **NRN1** | 0.1229 | **0.0371** | 0.1040 | **0.0666** | **0.0056** |
| **rs594734** | **6** | **5,962,211** | **NRN1** | 0.2715 | 0.2244 | **0.0626** | 0.2921 | 0.1816 |
| **rs11971348** | **7** | **98,090,205** | **NPTX2** | 0.2841 | 0.9042 | 0.6059 | 0.2924 | 0.5475 |
| **rs1728987** | **7** | **98,098,162** | **NPTX2** | **0.0522** | 0.4052 | 0.5673 | 0.2883 | 0.2633 |
| **rs6465693** | **7** | **98,104,773** | **NPTX2** | **0.0565** | **0.0742** | 0.6531 | 0.2258 | **0.0192** |
| **rs1434278** | **8** | **103,724,969** | **KLF10** | 0.4889 | 0.6383 | 0.4470 | 0.4939 | 0.3530 |
| **rs2436969** | **8** | **103,726,284** | **KLF10** | 0.2904 | 0.2217 | 0.1829 | 0.5941 | **0.0268** |
| **rs980112** | **8** | **103,734,688** | **KLF10** | 0.1105 | **0.0690** | **0.0973** | 0.1394 | 0.6652 |
| **rs2511730** | **8** | **103,739,213** | **KLF10** | 0.8747 | 0.2938 | 0.3506 | 0.7598 | 0.7353 |
| **rs11167152** | **8** | **143,688,078** | **Arc** | **0.0466** | **0.0438** | **0.0245** | 0.3993 | 0.4190 |
| **rs10097505** | **8** | **143,691,185** | **Arc** | 0.7594 | 0.5600 | 0.2023 | 0.1266 | 0.4003 |
| **rs1519479** | **11** | **27,624,106** | **BDNF** | 0.1563 | 0.1095 | **0.0985** | 0.9513 | 0.1701 |
| **rs6265** | **11** | **27,636,491** | **BDNF** | 0.5314 | 0.5872 | 0.2403 | **0.0964** | 0.8408 |
| **rs12273363** | **11** | **27,701,434** | **BDNF** | **0.0407** | 0.1247 | **0.0392** | 0.2361 | 0.1936 |
| **rs908867** | **11** | **27,702,339** | **BDNF** | 0.5009 | 0.8552 | 0.5075 | 0.9123 | 0.5093 |
| **rs1491850** | **11** | **27,706,300** | **BDNF** | **0.0161** | **0.0611** | **0.0132** | 0.5408 | 0.5123 |
| **rs1491851** | **11** | **27,709,338** | **BDNF** | 0.2007 | 0.2895 | 0.3954 | **0.0793** | **0.0739** |
| **rs10492555** | **13** | **35,607,108** | **DCLK1** | 0.2193 | **0.0569** | 0.1294 | 0.4667 | **0.0437** |
| **rs9315390** | **13** | **35,606,999** | **DCLK1** | 0.1308 | 0.1537 | 0.3419 | 0.3973 | 0.6026 |
| **rs1001232** | **13** | **35,606,207** | **DCLK1** | **0.0238** | **0.0560** | 0.1649 | **0.0704** | 0.3086 |
| **rs1926324** | **13** | **35,582,442** | **DCLK1** | 0.5159 | 0.6196 | 0.5640 | 0.4760 | 0.6508 |
| **rs6563330** | **13** | **35,557,362** | **DCLK1** | 0.5403 | 0.7445 | 0.4795 | 0.6641 | 0.1741 |
| **rs9315383** | **13** | **35,549,854** | **DCLK1** | **0.0034** | **0.0370** | **0.0561** | **0.0112** | **0.0469** |
| **rs7334245** | **13** | **35,545,152** | **DCLK1** | **0.0060** | **0.0423** | 0.1435 | **0.0460** | 0.1003 |
| **rs1926317** | **13** | **35,542,039** | **DCLK1** | **0.0246** | 0.1862 | 0.1482 | 0.1938 | 0.1800 |
| **rs7989807** | **13** | **35,523,088** | **DCLK1** | **0.0956** | **0.0416** | **0.0614** | 0.2180 | 0.5427 |
| **rs7323560** | **13** | **35,510,464** | **DCLK1** | 0.2028 | 0.3213 | **0.0730** | **0.0014** | 0.3401 |
| **rs8000458** | **13** | **35,419,986** | **DCLK1** | 0.7452 | 0.2947 | 0.5249 | **0.0685** | 0.3238 |
| **rs7989245** | **13** | **35,397,596** | **DCLK1** | 0.1535 | 0.1060 | 0.2688 | 0.1013 | **0.0061** |
| **rs1926452** | **13** | **35,342,936** | **DCLK1** | **0.0120** | **0.0765** | **0.0295** | **0.0171** | 0.1451 |
| **rs10507435** | **13** | **35,338,995** | **DCLK1** | **0.0021** | **0.0039** | **0.00043** | **0.0204** | **0.0023** |
| **rs943220** | **13** | **35,337,251** | **DCLK1** | **0.0036** | **0.00060** | **0.00010** | 0.2563 | **0.0054** |
| **rs2148417** | **13** | **35,331,861** | **DCLK1** | 0.3754 | 0.1641 | 0.2618 | 0.6361 | 0.3820 |
| **rs4391923** | **13** | **35,328,855** | **DCLK1** | 0.6138 | 0.1845 | 0.3099 | **0.0932** | **0.0027** |
| **rs1926466** | **13** | **35,326,594** | **DCLK1** | 0.3661 | 0.1764 | 0.3341 | 0.7720 | 0.1390 |
| **rs9574707** | **13** | **35,301,834** | **DCLK1** | 0.1573 | **0.0988** | **0.0346** | 0.3711 | 0.1092 |
| **rs2296645** | **13** | **35,300,425** | **DCLK1** | 0.1417 | 0.6333 | 0.1936 | **0.0641** | 0.2171 |
| **rs1926467** | **13** | **35,291,374** | **DCLK1** | 0.3869 | 0.2740 | 0.1247 | **0.0025** | 0.5896 |
| **rs7328001** | **13** | **35,271,238** | **DCLK1** | 0.1899 | 0.3022 | 0.3899 | 0.4158 | 0.2577 |
| **rs12430800** | **13** | **35,257,973** | **DCLK1** | **0.0977** | 0.2547 | 0.1495 | 0.3325 | 0.4433 |
| **rs4591003** | **13** | **35,256,049** | **DCLK1** | 0.1362 | 0.3244 | **0.0527** | **0.0045** | 0.6654 |
| **rs9545332** | **13** | **35,253,679** | **DCLK1** | 0.5737 | 0.4649 | 0.6212 | **0.0267** | 0.6190 |
| **rs872060** | **13** | **35,236,916** | **DCLK1** | 0.7194 | 0.5313 | 0.5707 | **0.0683** | 0.3682 |

Bonferroni correction p-value threshold = 0.001 (Note that the Bonferroni correction takes only the number of markers tested, not the number of variables, as these variables are not independent).

**Color legend for all statistical analyses:**

**Light blue: trend towards association, p-value= 0.1 to 0.05**

**Yellow p-value = 0.05 to 0.01**

**Orange p-value = 0.01 to 0.001**

**Purple p-value below 0.001**

### SOM Table 3. Single marker regression analysis in the NCNG sample.

Linear regression with each variable analyzed as the outcome predicted by the allele. Sex and age were used as covariates (same variables as for table 2).

| **SNP id** | **chr** | **position** | **Gene** | **CVLT Learning** | **CVLT 5 min free recall** | **CVLT 30 min free recall** | **WASI IQ** | **Years of education** |
| --- | --- | --- | --- | --- | --- | --- | --- | --- |
| **rs2038640** | **6** | **5 933 747** | **NRN1** | 0,7690 | 0,6351 | 0,3442 | 0,8516 | 0,6862 |
| **rs17363382** | **6** | **5 935 822** | **NRN1** | 0,7881 | 0,8531 | 0,8290 | 0,2631 | 0,3578 |
| **rs10484319** | **6** | **5 938 995** | **NRN1** | 0,9869 | 0,7984 | 0,9290 | 0,5310 | 0,2995 |
| **rs12333117** | **6** | **5 939 990** | **NRN1** | 0,3272 | 0,6482 | 0,8167 | 0,7841 | 0,8400 |
| **rs582186** | **6** | **5 946 379** | **NRN1** | 0,5882 | 0,7171 | 0,5769 | 0,8304 | 0,2419 |
| **rs3763180** | **6** | **5 954 846** | **NRN1** | 0,6915 | 0,8226 | 0,8766 | 0,6768 | 0,8297 |
| **rs594734** | **6** | **5 962 211** | **NRN1** | 0,2492 | 0,8153 | 0,7448 | 0,3258 | 0,1898 |
| **rs11971348** | **7** | **98 090 205** | **NPTX2** | 0,7990 | 0,8406 | 0,9353 | 0,6141 | 0,5227 |
| **rs1728987** | **7** | **98 098 162** | **NPTX2** | **0,0541** | 0,4475 | 0,7182 | 0,8294 | 0,2933 |
| **rs6465693** | **7** | **98 104 773** | **NPTX2** | **0,0858** | 0,1517 | 0,9349 | 0,1757 | 0,3358 |
| **rs1434278** | **8** | **103 724 969** | **KLF10** | 0,8835 | 0,8684 | 0,7812 | 0,7591 | 0,2855 |
| **rs2436969** | **8** | **103 726 284** | **KLF10** | 0,5232 | 0,4571 | 0,3303 | 0,5962 | 0,2228 |
| **rs980112** | **8** | **103 734 688** | **KLF10** | 0,7417 | 0,6218 | 0,4779 | 0,3774 | 0,6416 |
| **rs2511730** | **8** | **103 739 213** | **KLF10** | 0,6420 | 0,7006 | 0,6684 | 0,6647 | 0,7922 |
| **rs11167152** | **8** | **143 688 078** | **Arc** | 0,5862 | 0,8115 | 0,2013 | 0,6642 | 0,3342 |
| **rs10097505** | **8** | **143 691 185** | **Arc** | 0,9571 | 0,2679 | **0,0568** | 0,8695 | 0,4437 |
| **rs1519479** | **11** | **27 624 106** | **BDNF** | 0,1029 | 0,3352 | 0,2067 | 0,9286 | 0,5934 |
| **rs6265** | **11** | **27 636 491** | **BDNF** | 0,4645 | 0,8774 | 0,5728 | 0,6040 | 0,9808 |
| **rs12273363** | **11** | **27 701 434** | **BDNF** | **0,0337** | 0,2001 | **0,0719** | 0,6318 | 0,9281 |
| **rs908867** | **11** | **27 702 339** | **BDNF** | 0,8222 | 0,6521 | 0,9435 | 0,7982 | 0,3417 |
| **rs1491850** | **11** | **27 706 300** | **BDNF** | **0,0115** | **0,0678** | **0,0105** | 0,9397 | 0,9745 |
| **rs1491851** | **11** | **27 709 338** | **BDNF** | 0,5858 | 0,3916 | 0,7300 | 0,7233 | 0,9556 |
| **rs10492555** | **13** | **35 607 108** | **DCLK1** | 0,6307 | 0,1989 | 0,4351 | 0,4264 | **0,0694** |
| **rs9315390** | **13** | **35 606 999** | **DCLK1** | 0,5675 | 0,8091 | 0,5501 | 0,5093 | 0,7064 |
| **rs1001232** | **13** | **35 606 207** | **DCLK1** | **0,0135** | 0,1181 | 0,1642 | **0,0398** | 0,2086 |
| **rs1926324** | **13** | **35 582 442** | **DCLK1** | 0,4167 | 0,4926 | 0,4347 | 0,6491 | 0,5984 |
| **rs6563330** | **13** | **35 557 362** | **DCLK1** | 0,4524 | 0,9443 | 0,5885 | 0,7847 | 0,7514 |
| **rs9315383** | **13** | **35 549 854** | **DCLK1** | **0,0247** | 0,1173 | **0,0481** | **0,0045** | **0,0607** |
| **rs7334245** | **13** | **35 545 152** | **DCLK1** | **0,0452** | **0,0924** | 0,1768 | **0,0580** | **0,0788** |
| **rs1926317** | **13** | **35 542 039** | **DCLK1** | 0,3924 | 0,7479 | 0,2197 | 0,3604 | 0,3035 |
| **rs7989807** | **13** | **35 523 088** | **DCLK1** | 0,3595 | 0,2471 | 0,9205 | 0,3591 | 0,9275 |
| **rs7323560** | **13** | **35 510 464** | **DCLK1** | 0,1201 | 0,2118 | **0,0880** | 0,7493 | 0,9417 |
| **rs8000458** | **13** | **35 419 986** | **DCLK1** | 0,8892 | 0,2801 | 0,9771 | **0,0808** | 0,1268 |
| **rs7989245** | **13** | **35 397 596** | **DCLK1** | 0,6769 | 0,9433 | 0,9031 | 0,4021 | **0,0054** |
| **rs1926452** | **13** | **35 342 936** | **DCLK1** | 0,8872 | 0,5453 | 0,3555 | 0,9808 | **0,0975** |
| **rs10507435** | **13** | **35 338 995** | **DCLK1** | 0,2121 | **0,0803** | **0,0428** | **0,0271** | **0,0039** |
| **rs943220** | **13** | **35 337 251** | **DCLK1** | **0,0730** | **0,0209** | **0,0181** | 0,1393 | **0,0052** |
| **rs2148417** | **13** | **35 331 861** | **DCLK1** | 0,4117 | 0,1733 | 0,2855 | 0,5996 | 0,3236 |
| **rs4391923** | **13** | **35 328 855** | **DCLK1** | 0,9727 | 0,2141 | 0,2285 | 0,3361 | **0,0014** |
| **rs1926466** | **13** | **35 326 594** | **DCLK1** | 0,3263 | 0,2167 | 0,3094 | 0,7369 | **0,0676** |
| **rs9574707** | **13** | **35 301 834** | **DCLK1** | 0,7872 | 0,4113 | 0,1714 | 0,9610 | 0,7155 |
| **rs2296645** | **13** | **35 300 425** | **DCLK1** | 0,5103 | 0,6928 | 0,2868 | 0,1564 | 0,7921 |
| **rs1926467** | **13** | **35 291 374** | **DCLK1** | 0,5130 | 0,9691 | 0,5000 | **0,0250** | 0,4469 |
| **rs7328001** | **13** | **35 271 238** | **DCLK1** | 0,3348 | 0,8691 | 0,9449 | 0,4766 | 0,7541 |
| **rs12430800** | **13** | **35 257 973** | **DCLK1** | 0,5305 | 0,7332 | 0,7106 | 0,8844 | 0,5857 |
| **rs4591003** | **13** | **35 256 049** | **DCLK1** | 0,4882 | 0,9160 | 0,6113 | **0,0786** | 0,8554 |
| **rs9545332** | **13** | **35 253 679** | **DCLK1** | 0,7289 | 0,5913 | 0,7685 | 0,1355 | 0,4885 |
| **rs872060** | **13** | **35 236 916** | **DCLK1** | 0,6211 | 0,4353 | 0,5275 | 0,1115 | 0,6352 |

### SOM table 4: Haplotype trend regression in the NCNG sample.

Results from the haplotype trend regressions 2- (HTR2) and 3- (HTR3) markers sliding window, performed with all the markers genotyped in the NCNG sample. All regressions were performed using sex and age as covariates.

|  |  |  | **CVLTII learning** | | **CVLTII 5 min delay recall** | | **CVLTII 30 min delay recall** | | **Years of education** | | **WASI IQ** |  |
| --- | --- | --- | --- | --- | --- | --- | --- | --- | --- | --- | --- | --- |
| **SNP id** | **chr** | **Gene** | **htr2** | **htr3** | **htr2** | **htr3** | **htr2** | **htr3** | **htr2** | **htr3** | **htr2** | **htr3** |
| **rs2038640** | **6** | **NRN1** | 0.8128 | 0.8347 | 0.8206 | 0.5599 | 0.6750 | 0.8411 | 0.5851 | **0.0605** | 0.4098 | 0.8452 |
| **rs17363382** | **6** | **NRN1** | 0.9807 | 0.8876 | 0.6466 | 0.7382 | 0.9610 | 0.9527 | 0.2052 | 0.2704 | 0.6570 | 0.7180 |
| **rs10484319** | **6** | **NRN1** | 0.8055 | 0.6406 | 0.9093 | 0.9375 | 0.8943 | 0.9892 | 0.4617 | 0.6725 | 0.8187 | 0.9407 |
| **rs12333117** | **6** | **NRN1** | 0.2866 | 0.4778 | 0.4784 | 0.8333 | 0.6285 | 0.8958 | 0.5712 | 0.3567 | 0.5019 | 0.8566 |
| **rs582186** | **6** | **NRN1** | 0.6912 | 0.3688 | 0.9808 | 0.6791 | 0.8983 | 0.6852 | 0.1824 | 0.5810 | 0.9307 | 0.6299 |
| **rs3763180** | **6** | **NRN1** | 0.2316 |  | 0.2792 |  | 0.7226 |  | 0.7127 |  | 0.4577 |  |
| **rs594734** | **6** | **NRN1** |  |  |  |  |  |  |  |  |  |  |
| **rs11971348** | **7** | **NPTX2** | 0.1496 | 0.1831 | 0.7198 | 0.6477 | 0.9585 | 0.7250 | 0.6373 | 0.7200 | 0.6712 | 0.6969 |
| **rs1728987** | **7** | **NPTX2** | 0.1586 |  | 0.4015 |  | 0.8045 |  | 0.1999 |  | 0.3283 |  |
| **rs6465693** | **7** | **NPTX2** |  |  |  |  |  |  |  |  |  |  |
| **rs1434278** | **8** | **KLF10** | 0.8322 | 0.8179 | 0.7672 | 0.7754 | 0.6789 | 0.7114 | 0.2140 | 0.2558 | 0.6694 | 0.6168 |
| **rs2436969** | **8** | **KLF10** | 0.4751 | 0.3844 | 0.5101 | 0.6696 | 0.3428 | 0.6273 | 0.3399 | 0.3245 | 0.5751 | 0.7746 |
| **rs980112** | **8** | **KLF10** | 0.8603 |  | 0.8240 |  | 0.7227 |  | 0.8592 |  | 0.5934 |  |
| **rs2511730** | **8** | **KLF10** |  |  |  |  |  |  |  |  |  |  |
| **rs11167152** | **8** | **Arc** | 0.4614 |  | 0.2850 |  | 0.1473 |  | 0.7381 |  | 0.4328 |  |
| **rs10097505** | **8** | **Arc** |  |  |  |  |  |  |  |  |  |  |
| **rs1519479** | **11** | **BDNF** | 0.4021 | 0.2560 | 0.7925 | 0.5439 | 0.6618 | 0.2691 | 0.8390 | 0.9891 | 0.7929 | 0.5966 |
| **rs6265** | **11** | **BDNF** | 0.1201 | 0.1632 | 0.6127 | 0.5730 | 0.2560 | 0.3327 | 0.9991 | 0.8717 | 0.5686 | 0.8469 |
| **rs12273363** | **11** | **BDNF** | 0.1617 | **0.0933** | 0.4714 | 0.3520 | 0.3018 | 0.1147 | 0.6476 | 0.9110 | 0.9343 | **0.0551** |
| **rs908867** | **11** | **BDNF** | **0.0417** | 0.2005 | 0.1595 | 0.5291 | **0.0334** | 0.1556 | 0.8461 | 0.1892 | 0.9963 | 0.8199 |
| **rs1491850** | **11** | **BDNF** | 0.1059 |  | 0.3485 |  | **0.0848** |  | 0.4538 |  | 0.8429 |  |
| **rs1491851** | **11** | **BDNF** |  |  |  |  |  |  |  |  |  |  |
| **rs10492555** | **13** | **DCLK1** | 0.7469 | **0.0460** | 0.5857 | 0.2384 | 0.7707 | 0.4790 | 0.1782 | 0.1871 | 0.8048 | 0.1703 |
| **rs9315390** | **13** | **DCLK1** | **0.0525** | **0.0387** | 0.4829 | 0.4319 | 0.5739 | 0.5845 | 0.6461 | **0.0576** | 0.2357 | 0.2239 |
| **rs1001232** | **13** | **DCLK1** | **0.0570** | 0.1472 | 0.2845 | 0.6295 | 0.4019 | 0.4790 | 0.2396 | 0.4393 | 0.1201 | **0.0507** |
| **rs1926324** | **13** | **DCLK1** | 0.6598 | **0.0026** | 0.8866 | 0.1433 | 0.1612 | **0.0166** | 0.9404 | 0.1014 | 0.1360 | **0.0027** |
| **rs6563330** | **13** | **DCLK1** | **0.0031** | **0.0134** | 0.1940 | 0.1802 | **0.0482** | 0.1263 | 0.2103 | 0.1904 | **0.0087** | **0.0068** |
| **rs9315383** | **13** | **DCLK1** | **0.0672** | **0.0720** | 0.3149 | 0.1105 | 0.3458 | 0.4346 | 0.2297 | 0.2832 | **0.0522** | **0.0058** |
| **rs7334245** | **13** | **DCLK1** | **0.0677** | **0.0181** | 0.1088 | **0.0957** | 0.2778 | 0.1622 | 0.1703 | 0.3558 | **0.0223** | **0.0077** |
| **rs1926317** | **13** | **DCLK1** | 0.3239 | 0.2780 | 0.5604 | 0.3855 | 0.4792 | 0.2042 | 0.7478 | 0.6743 | 0.7184 | 0.8346 |
| **rs7989807** | **13** | **DCLK1** | 0.3028 | 0.5827 | 0.2892 | 0.3081 | 0.2456 | 0.7297 | 0.9699 | 0.3679 | 0.6831 | 0.5818 |
| **rs7323560** | **13** | **DCLK1** | 0.2289 | 0.8790 | **0.0969** | 0.8757 | 0.2058 | 0.6804 | 0.3894 | **0.0093** | 0.3250 | 0.3898 |
| **rs8000458** | **13** | **DCLK1** | 0.9304 | 0.6926 | 0.4737 | 0.9551 | 0.8121 | 0.9180 | **0.0212** | **0.0154** | 0.3663 | 0.5918 |
| **rs7989245** | **13** | **DCLK1** | 0.9360 | 0.5417 | 0.6858 | 0.2456 | 0.5539 | 0.2073 | **0.0130** | **0.0030** | 0.8686 | 0.1069 |
| **rs1926452** | **13** | **DCLK1** | **0.0098** | 0.2803 | **0.0808** | 0.1900 | **0.0357** | 0.1849 | **0.0277** | **0.0322** | **0.0023** | **0.0353** |
| **rs10507435** | **13** | **DCLK1** | 0.3658 | 0.2946 | 0.1487 | **0.0530** | 0.1323 | **0.0578** | **0.0154** | **0.0144** | 0.1838 | 0.1721 |
| **rs943220** | **13** | **DCLK1** | 0.1645 | 0.3378 | **0.0310** | **0.0449** | **0.0295** | **0.0464** | **0.0080** | **0.0003** | 0.2711 | 0.2263 |
| **rs2148417** | **13** | **DCLK1** | 0.5676 | 0.7908 | 0.1236 | 0.3534 | 0.2417 | 0.4709 | **0.0046** | **0.0316** | 0.2620 | 0.5515 |
| **rs4391923** | **13** | **DCLK1** | 0.3386 | 0.8618 | 0.3306 | 0.7317 | 0.1463 | 0.5914 | **0.0291** | **0.0251** | 0.2169 | 0.8518 |
| **rs1926466** | **13** | **DCLK1** | 0.7898 | 0.7507 | 0.5851 | 0.5948 | 0.6588 | 0.6186 | **0.0675** | **0.0849** | 0.9705 | 0.6654 |
| **rs9574707** | **13** | **DCLK1** | 0.6524 | 0.9066 | 0.7848 | 0.9037 | 0.3005 | 0.4085 | 0.2537 | 0.7408 | 0.3037 | 0.1401 |
| **rs2296645** | **13** | **DCLK1** | 0.8214 | 0.5988 | 0.9004 | 0.8503 | 0.5959 | 0.7425 | 0.8153 | 0.9192 | **0.0621** | 0.1069 |
| **rs1926467** | **13** | **DCLK1** | 0.3591 | 0.4352 | 0.4439 | 0.9768 | 0.6489 | 0.8871 | 0.7227 | 0.5312 | 0.1357 | **0.0215** |
| **rs7328001** | **13** | **DCLK1** | 0.4394 | 0.7215 | 0.7219 | 0.9875 | 0.8439 | 0.8637 | 0.6574 | 0.8286 | 0.5857 | **0.0893** |
| **rs12430800** | **13** | **DCLK1** | 0.1386 | 0.5415 | 0.5843 | 0.7843 | 0.1843 | 0.7381 | 0.8923 | 0.9550 | **0.0467** | **0.0190** |
| **rs4591003** | **13** | **DCLK1** | 0.6487 | 0.5138 | 0.8932 | 0.7032 | 0.7011 | 0.5946 | 0.8306 | 0.6206 | 0.1254 | **0.0814** |
| **rs9545332** | **13** | **DCLK1** | 0.6371 |  | 0.5777 |  | 0.6144 |  | 0.5358 |  | 0.1267 |  |
| **rs872060** | **13** | **DCLK1** |  |  |  |  |  |  |  |  |  |  |

### SOM table 5: HTR with LBC markers in the NCNG sample.

Results from the haplotype trend regressions 2- (HTR2) and 3- (HTR3) markers sliding window, performed only for the *DCLK1* markers genotyped in the LBC samples (for comparison between the samples).

| **Variable** | **CVLTII learning** | | **CVLTII 5 min delay recall** | | **CVLTII 30 min delay recall** | | **Years of education** | | **WASI IQ** | |
| --- | --- | --- | --- | --- | --- | --- | --- | --- | --- | --- |
|  | **HTR2** | **HTR3** | **HTR2** | **HTR3** | **HTR2** | **HTR3** | **HTR2** | **HTR3** | **HTR2** | **HTR3** |
| **rs10492555** | 0.7469 | 0.1397 | 0.5857 | 0.4384 | 0.7707 | 0.1296 | 0.1782 | **0.0739** | 0.8048 | **0.0335** |
| **rs9315390** | **0.0265** | 0.1091 | 0.1628 | 0.2363 | **0.0948** | 0.2416 | 0.1280 | **0.0333** | **0.0063** | **0.0104** |
| **rs9315383** | **0.0672** | **0.0332** | 0.3149 | 0.2013 | 0.3458 | 0.2164 | 0.2297 | 0.3496 | **0.0522** | 0.1372 |
| **rs7334245** | **0.0053** | 0.1190 | **0.0507** | 0.3409 | 0.1806 | 0.2889 | 0.2599 | 0.5161 | **0.0641** | 0.2225 |
| **rs7989807** | 0.4882 | 0.9147 | 0.4134 | 0.8583 | 0.3839 | 0.6668 | 0.8072 | **0.0975** | 0.6521 | 0.6968 |
| **rs7323560** | 0.4729 | 0.7733 | 0.5633 | 0.4558 | 0.2389 | 0.1143 | **0.0078** | **0.0039** | 0.3884 | 0.3011 |
| **rs7989245** | 0.6787 | 0.7361 | 0.4843 | 0.4613 | 0.3073 | 0.4594 | **0.0011** | **0.0018** | 0.1026 | **0.0953** |
| **rs10507435** | 0.3658 | 0.7907 | 0.1487 | 0.2522 | 0.1323 | 0.3834 | **0.0154** | **0.0015** | 0.1838 | 0.1146 |
| **rs943220** | 0.3402 | 0.5864 | **0.0533** | 0.1021 | 0.1013 | 0.1136 | **0.0002** | **0.0044** | 0.1232 | 0.3809 |
| **rs4391923** | 0.6760 | 0.5460 | 0.2681 | 0.4180 | 0.2250 | 0.3992 | **0.0071** | **0.0217** | 0.5172 | 0.1678 |
| **rs2296645** | 0.8214 | 0.6866 | 0.9004 | 0.8052 | 0.5959 | 0.3992 | 0.8153 | 0.6850 | **0.0621** | **0.0460** |
| **rs1926467** | 0.3489 | 0.8687 | 0.6711 | 0.9983 | 0.2867 | 0.9517 | 0.5223 | 0.6170 | **0.0360** | 0.1040 |
| **rs12430800** | 0.1386 | 0.5415 | 0.5843 | 0.7843 | 0.1843 | 0.7381 | 0.8923 | 0.9550 | **0.0467** | **0.0190** |
| **rs4591003** | 0.6487 | 0.7045 | 0.8932 | 0.8622 | 0.7011 | 0.7973 | 0.8306 | 0.8299 | 0.1254 | 0.1169 |
| **rs9545332** | 0.8124 |  | 0.7808 |  | 0.8086 |  | 0.7378 |  | 0.1681 |  |
| **rs872060** |  |  |  |  |  |  |  |  |  |  |

### SOM Table 6: Two genetic loci interaction analyses for the CVLT II-30 min delayed recall trait, in the NCNG sample.

Two genetic loci interactions were performed with the Helix Tree software, 1326 possible interactions were tested for the trait: CVLTII_ 30 minutes delayed recall. Only the interactions significant after Bonferroni correction are displayed.

| **Marker 1** | **Marker 2** | **p-value** | **Bonferroni p-value** |
| --- | --- | --- | --- |
| **rs943220** | rs10492555 | 5.24E-07 | 0.00313552 |
| **rs11167152** | rs943220 | 1.95E-06 | 0.01552566 |
| **rs10507435** | rs10492555 | 5.52E-06 | 0.02944693 |
| **rs1491850** | rs10507435 | 4.04E-06 | 0.0334272 |

Markers info: rs11167152, marker tagging the ARC gene, rs1491850, marker tagging the BDNF gene, rs10492555, located 4 kb from 5’ of *DCLK1*, rs10507435 and rs943220, located within intron 5 of *DCLK1* gene.

## Data from all DCLK1 markers typed in the LBC1921

### SOM Table 7: Markers statistics in the LBC1921

**SNPids** are according to dbSNP ( <http://www.ncbi.nlm.nih.gov/projects/SNP/> ) reference sequence entries. **Chr** and **position**: chromosomal position of the SNP according to NCBI36 build, with the name of the **gene** that the SNP (**D/d**) is tagging; **MAF**: Minor Allele Frequency; **CR:** Call Rate, i.e. Sequenom genotyping success rate; **HWE P**: Hardy Weinberg Equilibrium p-value. Genotypes (**DD, Dd, dd and failed**) or Allele (**D, d and failed**) are given in counts.

| **SNP id** | **chr** | **position** | **D** | **d** | **MAF** | **Call Rate** | **HWE P** | **DD** | **Dd** | **dd** | **Failed** | **D** | **d** | **Failed** |
| --- | --- | --- | --- | --- | --- | --- | --- | --- | --- | --- | --- | --- | --- | --- |
| **rs10492555** | **13** | **35,607,108** | A | C | 0.158 | 0.987903 | 0.8011 | 13 | 129 | 348 | 6 | 155 | 825 | 12 |
| **rs9315390** | **13** | **35,606,999** | T | C | 0.259 | 0.989919 | 0.4584 | 36 | 182 | 273 | 5 | 254 | 728 | 10 |
| **rs9315383** | **13** | **35,549,854** | G | C | 0.476 | 0.977823 | 0.2011 | 103 | 256 | 126 | 11 | 462 | 508 | 22 |
| **rs7334245** | **13** | **35,545,152** | T | C | 0.43 | 0.961694 | 0.0237 | 76 | 258 | 143 | 19 | 410 | 544 | 38 |
| **rs7989807** | **13** | **35,523,088** | A | G | 0.127 | 0.977823 | 0.1193 | 4 | 115 | 366 | 11 | 123 | 847 | 22 |
| **rs7323560** | **13** | **35,510,464** | C | A | 0.062 | 0.989919 | 0.1422 | 0 | 61 | 430 | 5 | 61 | 921 | 10 |
| **rs7989245** | **13** | **35,397,596** | C | T | 0.35 | 0.891129 | 0.209 | 48 | 213 | 181 | 54 | 309 | 575 | 108 |
| **rs10507435** | **13** | **35,338,995** | G | A | 0.249 | 0.981855 | 0.3722 | 34 | 175 | 278 | 9 | 243 | 731 | 18 |
| **rs943220** | **13** | **35,337,251** | A | G | 0.428 | 0.951613 | 0.6315 | 89 | 226 | 157 | 24 | 404 | 540 | 48 |
| **rs4391923** | **13** | **35,328,855** | G | A | 0.199 | 0.854839 | 0.7241 | 18 | 133 | 273 | 72 | 169 | 679 | 144 |
| **rs2296645** | **13** | **35,300,425** | G | A | 0.425 | 0.977823 | 0.5158 | 84 | 244 | 157 | 11 | 412 | 558 | 22 |
| **rs1926467** | **13** | **35,291,374** | A | G | 0.462 | 0.945565 | 0.3586 | 95 | 243 | 131 | 27 | 433 | 505 | 54 |
| **rs12430800** | **13** | **35,257,973** | T | C | 0.157 | 0.870968 | 0.088 | 6 | 124 | 302 | 64 | 136 | 728 | 128 |
| **rs4591003** | **13** | **35,256,049** | T | C | 0.498 | 0.97379 | 0.6164 | 117 | 247 | 119 | 13 | 481 | 485 | 26 |
| **rs9545332** | **13** | **35,253,679** | T | C | 0.218 | 0.97379 | 0.7808 | 22 | 167 | 294 | 13 | 211 | 755 | 26 |
| **rs872060** | **13** | **35,236,916** | C | T | 0.154 | 0.991935 | 0.6636 | 13 | 126 | 353 | 4 | 152 | 832 | 8 |

### SOM Table 8: Genotypic analysis in the LBC1921.

The markers were analyzed for association to the following traits:

- IQ score derived from Moray House Test at age 11 (**IQ11**)
- IQ score derived from Moray House Test at age 79 (**IQ79**)
- Wechsler Memory Scale (**WMS**)-Revised **Logical Memory** Test: **immediate**, **delayed** and **total**.
- Ravens Matrices and verbal fluency were also analyzed but did not show any association to DCLK1 markers.

|  |  |  |  | **WMS Logical Memory** | | |
| --- | --- | --- | --- | --- | --- | --- |
|  | Code | **IQ11** | **IQ79** | **Immediate** | **Delayed** | **Total** |
| **rs10492555** | m5’.1 | 0.2520 | 0.2260 | 0.4990 | 0.5540 | 0.5120 |
| **rs9315390** | m5’.2 | 0.5610 | 0.3030 | 0.1030 | **0.0330** | **0.0570** |
| **rs9315383** | m3.1 | 0.5230 | 0.9860 | **0.0540** | **0.0870** | **0.0650** |
| **rs7334245** | m3.2 | 0.6240 | 0.2660 | 0.6190 | 0.6980 | 0.6490 |
| **rs7989807** | m3.3 | 0.3380 | 0.1830 | 0.7720 | 0.7660 | 0.9250 |
| **rs7323560** | m3.4 | 0.8600 | 0.9910 | 0.7330 | 0.7380 | 0.9900 |
| **rs7989245** | m4 | 0.7550 | 0.1260 | 0.2570 | 0.3720 | 0.2960 |
| **rs10507435** | m5.1 | 0.6770 | 0.3720 | 0.4230 | 0.6220 | 0.5070 |
| **rs943220** | m5.2 | 0.2020 | 0.4460 | 0.3280 | **0.0810** | 0.1870 |
| **rs4391923** | m5.3 | 0.3310 | 0.8700 | 0.3120 | **0.0920** | 0.1570 |
| **rs2296645** | m11 | 0.1240 | 0.5490 | **0.0130** | **0.0067** | **0.0068** |
| **rs1926467** | m15 | 0.3820 | **0.0810** | 0.6650 | 0.7770 | 0.9510 |
| **rs12430800** | m19.1 | 0.1390 | **0.0007** | 0.5020 | 0.1820 | 0.2900 |
| **rs4591003** | m19.2 | 0.1360 | 0.1240 | 0.2720 | 0.4340 | 0.3330 |
| **rs9545332** | m19.3 | 0.8190 | 0.3170 | 0.1910 | 0.1180 | 0.1340 |
| **rs872060** | m3’ | 0.1190 | **0.0210** | 0.1200 | **0.0410** | **0.0610** |

### SOM Table 9: Linear regression analysis in the LBC1921.

Same variables as for the genotypic analysis were studied. The regressions were performed using sex and age (in days) as covariables. The analyses were also performed with or without the IQ at age 11 as covariable.

|  |  |  |  |  | **WMS Logical memory** | |  |  |  |  |
| --- | --- | --- | --- | --- | --- | --- | --- | --- | --- | --- |
| **Variable** | Code | **IQ11** | **IQ79** | **IQ79rIQ11** | **Immediate** | **Immediate rIQ11** | **Delayed** | **Delayed rIQ11** | **Total** | **Total rIQ11** |
| **rs10492555** | m5’.1 | 0.2680 | 0.1710 | 0.6300 | 0.6090 | 0.9450 | 0.6160 | 0.7320 | 0.5990 | 0.8780 |
| **rs9315390** | m5’.2 | 0.4840 | 0.6850 | 0.8480 | 0.9890 | 0.7760 | 0.6480 | 0.6730 | 0.7990 | 0.7100 |
| **rs9315383** | m3.1 | 0.8140 | 0.9640 | 0.4750 | 0.7760 | 0.7900 | 0.3110 | 0.4240 | 0.4910 | 0.7620 |
| **rs7334245** | m3.2 | 0.6870 | 0.3690 | **0.0750** | 0.5870 | 0.9910 | 0.6600 | 0.8100 | 0.6120 | 0.9000 |
| **rs7989807** | m3.3 | 0.2820 | 0.2860 | 0.2300 | 0.7280 | 0.6970 | 0.7890 | 0.9300 | 0.9780 | 0.8850 |
| **rs7323560** | m3.4 | 0.8420 | 0.9210 | 0.6060 | 0.7590 | 0.7390 | 0.7200 | 0.3030 | 0.9660 | 0.4670 |
| **rs7989245** | m4 | 0.9280 | 0.4520 | 0.7630 | 0.2630 | 0.6110 | 0.3130 | 0.7810 | 0.2710 | 0.6870 |
| **rs10507435** | m5.1 | 0.7810 | 0.5430 | 0.2870 | 0.3610 | 0.3860 | 0.9150 | 0.9860 | 0.6100 | 0.6750 |
| **rs943220** | m5.2 | 0.5800 | 0.8680 | 0.9260 | 0.7110 | 0.6850 | 0.1420 | **0.0950** | 0.3280 | 0.2660 |
| **rs4391923** | m5.3 | 0.3260 | 0.8410 | 0.8320 | 0.3070 | 0.3430 | 0.1410 | 0.2740 | 0.1930 | 0.2860 |
| **rs2296645** | m11 | 0.2330 | 0.4460 | 0.4380 | 0.4530 | 0.2910 | 0.6030 | 0.3470 | 0.5140 | 0.3000 |
| **rs1926467** | m15 | 0.8070 | **0.0660** | **0.0140** | 0.6730 | 0.7450 | 0.8230 | 0.5710 | 0.9300 | 0.8820 |
| **rs12430800** | m19.1 | 0.2560 | **0.0042** | **0.0019** | 0.5010 | 0.4080 | 0.1750 | 0.1030 | 0.2850 | 0.1940 |
| **rs4591003** | m19.2 | 0.8060 | 0.1470 | **0.0390** | 0.8530 | 0.9070 | 0.7390 | 0.6700 | 0.7850 | 0.7720 |
| **rs9545332** | m19.3 | 0.9650 | 0.3790 | 0.1850 | 0.7050 | 0.4580 | 0.4280 | 0.1780 | 0.5370 | 0.2700 |
| **rs872060** | m3’ | 0.1800 | **0.0260** | **0.0380** | 0.9460 | 0.8590 | 0.4300 | 0.3230 | 0.6940 | 0.6520 |

### SOM Table 10: Haplotype trend regression analysis in the LBC1921

Two- and three- markers sliding windows haplotype trend regression were performed for the IQ variables. Age and sex were used as covariables. Association to IQ79 was also tested using the IQ at age 11 as covariable.

|  |  | **IQ11** |  |  | **IQ79** |  |  | **IQ79-rIQ11** | |  |
| --- | --- | --- | --- | --- | --- | --- | --- | --- | --- | --- |
| **Variable** | Code | **LR** | **HTR2** | **HTR3** | **LR** | **HTR2** | **HTR3** | **LR** | **HTR2** | **HTR3** |
| **rs10492555** | m5’.1 | 0.26844 | 0.65002 | 0.41252 | 0.17094 | 0.57013 | 0.71834 | 0.63017 | 0.86618 | 0.91747 |
| **rs9315390** | m5’.2 | 0.48443 | 0.64978 | 0.87503 | 0.68518 | 0.93703 | 0.72572 | 0.84765 | 0.75670 | **0.05153** |
| **rs9315383** | m3.1 | 0.81365 | 0.14681 | 0.56372 | 0.96448 | **0.04048** | 0.76482 | 0.47488 | **0.07562** | 0.19049 |
| **rs7334245** | m3.2 | 0.68748 | 0.64491 | 0.39500 | 0.36926 | 0.61101 | 0.57634 | **0.07531** | 0.13162 | 0.27230 |
| **rs7989807** | m3.3 | 0.28186 | 0.39618 | 0.91063 | 0.28590 | 0.66620 | 0.94443 | 0.23002 | 0.28956 | 0.68887 |
| **rs7323560** | m3.4 | 0.84174 | 0.99666 | 0.89513 | 0.92071 | 0.93598 | 0.92080 | 0.60552 | 0.94526 | 0.46829 |
| **rs7989245** | m4 | 0.92774 | 0.79667 | 0.84450 | 0.45241 | 0.80177 | 0.64088 | 0.76279 | 0.45665 | **0.08929** |
| **rs10507435** | m5.1 | 0.78062 | 0.95539 | 0.79764 | 0.54347 | 0.54618 | 0.86874 | 0.28744 | 0.32715 | 0.40186 |
| **rs943220** | m5.2 | 0.57970 | 0.69953 | 0.69084 | 0.86826 | 0.96250 | 0.91219 | 0.92644 | 0.85349 | 0.85789 |
| **rs4391923** | m5.3 | 0.32597 | 0.61123 | 0.40839 | 0.84086 | 0.86913 | 0.58453 | 0.83215 | 0.88008 | 0.24500 |
| **rs2296645** | m11 | 0.23332 | 0.69147 | 0.28131 | 0.44581 | 0.17965 | **0.05730** | 0.43806 | **0.09336** | **0.02327** |
| **rs1926467** | m15 | 0.80664 | 0.57689 | 0.73645 | **0.06556** | **0.01577** | **0.05102** | **0.01447** | **0.00272** | **0.01365** |
| **rs12430800** | m19.1 | 0.25593 | 0.40123 | 0.35079 | **0.00424** | **0.02410** | **0.02234** | **0.00193** | **0.00508** | **0.00034** |
| **rs4591003** | m19.2 | 0.80604 | 0.49496 | 0.31510 | 0.14665 | **0.09104** | **0.01438** | **0.03866** | **0.00250** | **0.00281** |
| **rs9545332** | m19.3 | 0.96463 | 0.22312 |  | 0.37938 | **0.04799** |  | 0.18532 | 0.15371 |  |
| **rs872060** | m3’ | 0.18040 |  |  | **0.02622** |  |  | **0.03763** |  |  |

## Data from all DCLK1 markers typed in the LBC1936

### SOM Table 11: Marker statistics in the LBC1936.

**SNPids** are according to dbSNP ( <http://www.ncbi.nlm.nih.gov/projects/SNP/> ) reference sequence entries. **Chr** and **position**: chromosomal position of the SNP according to NCBI36 build, with the name of the **gene** that the SNP (**D/d**) is tagging; **MAF**: Minor Allele Frequency; **CR:** Call Rate, i.e. Sequenom genotyping success rate; **HWE P**: Hardy Weinberg Equilibrium p-value. Genotypes (**DD, Dd, dd and failed**) or Allele (**D, d and failed**) are given in counts.

| **SNP id** | **chr** | **position** | **D** | **d** | **MAF** | **CR** | **HWE P** | **DD** | **Dd** | **Dd** | **Failed** | **D** | **d** | **Failed** |
| --- | --- | --- | --- | --- | --- | --- | --- | --- | --- | --- | --- | --- | --- | --- |
| **rs872060** | **13** | **35,236,916** | C | T | 0.144 | 0.851 | 0.408 | 22 | 218 | 667 | 159 | 262 | 1552 | 318 |
| **rs9545332** | **13** | **35,253,679** | T | C | 0.214 | 0.977 | 0.038 | 59 | 328 | 655 | 24 | 446 | 1638 | 48 |
| **rs4591003** | **13** | **35,256,049** | T | C | 0.494 | 0.938 | 0.616 | 248 | 492 | 260 | 66 | 988 | 1012 | 132 |
| **rs12430800** | **13** | **35,257,973** | T | C | 0.162 | 0.963 | 0.035 | 36 | 260 | 731 | 39 | 332 | 1722 | 78 |
| **rs1926467** | **13** | **35,291,374** | A | G | 0.456 | 0.967 | 0.471 | 220 | 500 | 311 | 35 | 940 | 1122 | 70 |
| **rs2296645** | **13** | **35,300,425** | G | A | 0.449 | 0.957 | 0.291 | 214 | 488 | 318 | 46 | 916 | 1124 | 92 |
| **rs4391923** | **13** | **35,328,855** | G | A | 0.187 | 0.926 | 0.109 | 27 | 316 | 644 | 79 | 370 | 1604 | 158 |
| **rs943220** | **13** | **35,337,251** | A | G | 0.396 | 0.964 | 0.146 | 150 | 514 | 364 | 38 | 814 | 1242 | 76 |
| **rs10507435** | **13** | **35,338,995** | G | A | 0.249 | 0.977 | 0.083 | 54 | 410 | 577 | 25 | 518 | 1564 | 50 |
| **rs7989245** | **13** | **35,397,596** | C | T | 0.318 | 0.927 | 0.362 | 106 | 416 | 466 | 78 | 628 | 1348 | 156 |
| **rs7323560** | **13** | **35,510,464** | C | A | 0.059 | 0.977 | 0.838 | 4 | 115 | 922 | 25 | 123 | 1959 | 50 |
| **rs7989807** | **13** | **35,523,088** | A | G | 0.119 | 0.917 | 0.704 | 15 | 202 | 761 | 88 | 232 | 1724 | 176 |
| **rs7334245** | **13** | **35,545,152** | T | C | 0.422 | 0.955 | 0.065 | 196 | 468 | 354 | 48 | 860 | 1176 | 96 |
| **rs9315383** | **13** | **35,549,854** | G | C | 0.486 | 0.968 | 0.469 | 250 | 504 | 278 | 34 | 1004 | 1060 | 68 |
| **rs9315390** | **13** | **35,606,999** | T | C | 0.271 | 0.953 | 0.945 | 74 | 402 | 540 | 50 | 550 | 1482 | 100 |
| **rs10492555** | **13** | **35,607,108** | A | C | 0.138 | 0.983 | 0.187 | 25 | 239 | 784 | 18 | 289 | 1807 | 36 |

### SOM Table 12: Genotypic analysis in the LBC1936.

The following variables were analyzed for association to DCLK1 markers

| **blkdes** | WAIS III - Block Design total score. |
| --- | --- |
| **crtmean** | Four choice reaction time mean. |
| **crts.d.** | Four choice reaction time standard deviation. |
| **digback** | WAIS III - Digit Span Backwards. |
| **digsym** | WAIS III - Digit Symbol-coding total score. |
| **Gfactor** | G cognition (regression factor score) PCA WAIS. |
| **Gspeed** | G processing speed (regression factor score) PCA processing speed. |
| **IQ-11** | Age-11 IQ (age-11 MHT corrected for age in days at time of testing, then converted to IQ score) |
| **IQ-70** | Age-70 IQ. (age-70 MHT corrected for age in days at time of testing, then converted to IQ score) |
| **ittotal** | Inspection Time total correct responses. |
| **lm1re** | WMS III - Logical Memory I Total recall score (A + B + B2). |
| **lm1stre** | WMS III - Logical Memory I 1st recall total score (A + B). |
| **lm2re** | WMS III - Logical Memory II Delayed recall total score (A + B). |
| **lm slope** | WMS III - Logical Memory I Learning slope (B2 - B). |
| **lnseq** | WAIS III - Letter-Number Sequencing. |
| **matreas** | WAIS III - Matrix Reasoning total score. |
| **spanb** | WMS III - Spatial Span Backward. |
| **spanf** | WMS III - Spatial Span Forward. |
| **srt mean** | Simple reaction time mean. |
| **srts.d.** | Simple reaction time standard deviation. |
| **symsear** | WAIS III - Symbol Search. |
| **vftot** | Verbal fluency total score. |
| **vpa1st** | WMS III - Verbal Paired Associates I - 1st recall total score (list A). |
| **vpa2** | WMS III - Verbal Paired Associates II - recall total score. |
| **vpaslp** | WMS III - Verbal Paired Associates I - learning slope (List D - A). |

**MHT**: Moray House Test

**WAIS**: Wechsler Adult Intelligence Scale version III [1].

**WMS**: Wechsler Memory Scale version III [2].

There was no genotypic association (data not shown) to the following traits:

- Four choice reaction time mean and standard deviation
- Inspection total time correct responses
- Simple reaction Time mean and standard deviation
- Verbal fluency total score.

| **Variable** | Code | vpa slope | vpa 1st | vpa21 | sym sear | dig sym | digback | spanf | spanb | matreas | ln seq | blkdes | lm slope | lm1re | lm2re | gspeed | gfactor | iq11 | iq70 |
| --- | --- | --- | --- | --- | --- | --- | --- | --- | --- | --- | --- | --- | --- | --- | --- | --- | --- | --- | --- |
| **rs10492555** | m5’.1 | 0.1074 | **0.0967** | 0.2586 | 0.3485 | 0.9482 | **0.0229** | **0.0429** | 0.2269 | 0.2288 | 0.1269 | 0.1216 | **0.0920** | **0.0513** | **0.0327** | 0.6657 | 0.1758 | **0.0341** | **0.0220** |
| **rs9315390** | m5’.2 | **0.0853** | 0.2250 | 0.4869 | 0.4083 | 0.7400 | 0.6567 | 0.4342 | 0.2775 | 0.4951 | **0.0356** | 0.1570 | 0.5220 | 0.4646 | 0.7097 | 0.6724 | 0.3987 | 0.5010 | 0.2902 |
| **rs9315383** | m3.1 | 0.1035 | **0.0415** | 0.6633 | 0.4665 | 0.5141 | 0.8370 | **0.0461** | 0.5456 | 0.1961 | 0.5055 | 0.4676 | 0.6756 | 0.7882 | 0.6011 | 0.2707 | 0.4100 | 0.6771 | 0.3510 |
| **rs7334245** | m3.2 | **0.0312** | 0.5767 | **0.0831** | 0.3352 | 0.1312 | 0.6333 | **0.0508** | 0.1249 | 0.2804 | 0.6756 | 0.1057 | 0.7403 | 0.3791 | 0.5842 | 0.1139 | 0.2218 | 0.3308 | 0.3945 |
| **rs7989807** | m3.3 | 0.6481 | 0.4356 | 0.4170 | 0.7132 | 0.3285 | 0.8935 | 0.4460 | 0.5218 | 0.2130 | 0.8537 | 0.2157 | 0.6358 | 0.5619 | 0.5142 | 0.9463 | 0.3881 | 0.6157 | 0.6173 |
| **rs7323560** | m3.4 | 0.7126 | 0.2131 | 0.2450 | 0.5000 | 0.4683 | 0.3881 | 0.5417 | 0.8380 | 0.5278 | 0.2748 | 0.8582 | 0.8388 | 0.3760 | 0.1296 | 0.1918 | 0.8540 | 0.6660 | 0.5931 |
| **rs7989245** | m4 | 0.1382 | 0.7689 | 0.3363 | 0.6498 | 0.9233 | 0.3529 | 0.5205 | 0.1954 | 0.4353 | 0.8040 | 0.4208 | 0.3280 | 0.1712 | **0.0581** | 0.2643 | 0.6027 | 0.5463 | 0.4289 |
| **rs10507435** | m5.1 | 0.5022 | 0.4105 | **0.0652** | 0.6149 | 0.3003 | 0.5249 | 0.5350 | 0.2350 | 0.2631 | **0.0254** | 0.1235 | 0.4827 | 0.2043 | 0.1212 | 0.3902 | 0.4275 | 0.1781 | 0.2370 |
| **rs943220** | m5.2 | 0.4425 | 0.5236 | 0.6576 | 0.6190 | 0.2411 | **0.0344** | 0.7494 | 0.9403 | 0.1292 | 0.6537 | 0.3863 | 0.5460 | 0.3840 | 0.6056 | 0.1792 | 0.5785 | 0.3477 | 0.2606 |
| **rs4391923** | m5.3 | **0.0617** | 0.1355 | 0.4999 | **0.0234** | 0.2502 | 0.3210 | 0.1003 | **0.0133** | **0.0649** | **0.0871** | **0.0075** | **0.0067** | 0.1039 | **0.0967** | 0.1613 | **0.0208** | 0.1148 | **0.0119** |
| **rs2296645** | m11 | **0.0171** | 0.1102 | 0.4325 | **0.0549** | 0.1780 | 0.6718 | 0.3302 | 0.5545 | 0.3758 | **0.0534** | 0.4987 | 0.5150 | 0.1040 | 0.4874 | 0.3585 | 0.2554 | 0.2059 | 0.6082 |
| **rs1926467** | m15 | 0.1702 | 0.2962 | 0.8368 | **0.0590** | 0.2105 | **0.0669** | **0.0011** | 0.3551 | **0.0274** | **0.0334** | 0.2492 | 0.2024 | 0.4249 | 0.3625 | 0.1614 | **0.0296** | **0.0139** | **0.0613** |
| **rs12430800** | m19.1 | 0.4498 | 0.1600 | 0.4703 | 0.1904 | 0.4189 | 0.7373 | **0.0379** | 0.4885 | **0.0875** | 0.7414 | **0.0031** | 0.8644 | 0.5046 | 0.4335 | 0.1454 | 0.2353 | **0.0672** | **0.0656** |
| **rs4591003** | m19.2 | 0.5954 | 0.4153 | 0.7390 | 0.1479 | 0.2747 | 0.1517 | **0.0053** | 0.2780 | **0.0591** | 0.1275 | 0.1411 | 0.3401 | 0.4571 | 0.2306 | 0.4362 | **0.0845** | **0.0371** | 0.3446 |
| **rs9545332** | m19.3 | **0.0335** | 0.5647 | **0.0212** | 0.1309 | **0.0257** | 0.4081 | 0.4790 | 0.4251 | 0.2501 | **0.0164** | **0.0307** | **0.0922** | 0.1192 | **0.0186** | **0.0251** | **0.0212** | **0.0178** | 0.2442 |
| **rs872060** | m3’ | **0.0582** | 0.5677 | 0.3375 | 0.5984 | 0.5128 | 0.2977 | 0.8178 | 0.4185 | 0.4487 | 0.3056 | 0.3082 | 0.3482 | 0.3236 | **0.0722** | 0.3580 | 0.3657 | 0.5934 | 0.9102 |
|  |  |  |  |  |  |  |  |  |  |  |  |  |  |  |  |  |  |  |  |

### SOM Table 13: Linear regression analysis in the LBC1936.

Same variables as for the genotypic analysis were studied (SOM table 12). The regressions were performed using sex and age (in days) as covariables. The analyses were performed also with or without the IQ at age 11 as covariable (regression with IQ11 is displayed only if there is significant association i.e. p-value < 0.05).

There was no genotypic association (data not shown) to the following traits:

- Inspection total time correct responses
- Verbal fluency total score.

| **Variable** | **IQ-11** | **IQ-70** | **IQ-70 rIQ-11** | **Gfactor** | **Gfactor rIQ-11** | **Gspeed** |
| --- | --- | --- | --- | --- | --- | --- |
| **rs10492555** | **0,058** | 0,90 | 0,38 | 0,70 | 0,62 | 0,87 |
| **rs9315390** | 0,52 | 0,68 | 0,74 | 0,79 | 0,46 | 0,88 |
| **rs9315383** | 0,67 | 0,92 | 0,98 | 0,66 | 0,57 | 0,34 |
| **rs7334245** | 0,81 | 0,47 | 0,41 | 0,19 | **0,089** | 0,18 |
| **rs7989807** | 0,93 | 0,57 | 0,52 | 0,77 | 0,82 | 0,67 |
| **rs7323560** | 0,71 | 0,59 | 0,64 | 0,79 | 0,81 | 0,16 |
| **rs7989245** | 0,82 | 0,81 | 0,37 | 0,62 | 0,21 | 0,22 |
| **rs10507435** | 0,65 | 0,48 | 0,85 | 0,48 | 0,91 | 0,88 |
| **rs943220** | 0,39 | 0,32 | 0,57 | 0,51 | 0,87 | 0,97 |
| **rs4391923** | 0,15 | **0,010** | **0,027** | **0,018** | **0,046** | 0,11 |
| **rs2296645** | 0,37 | 0,60 | **0,083** | 0,46 | **0,059** | 0,33 |
| **rs1926467** | **0,023** | 0,13 | 0,57 | **0,027** | 0,16 | 0,35 |
| **rs12430800** | 0,55 | 0,68 | 0,88 | 0,56 | 0,62 | 0,12 |
| **rs4591003** | **0,015** | 0,27 | 0,77 | **0,064** | 0,46 | 0,70 |
| **rs9545332** | **0,023** | 0,21 | 0,75 | **0,034** | 0,25 | **0,034** |
| **rs872060** | 0,85 | 0,99 | 0,76 | 0,43 | 0,39 | 0,64 |

| **Variable** | **crt s.d.** | **crts.d. rIQ11** | **lm slope1** | **lmslop rIQ11** | **lm 2re** | **vpaslp** | **vpaslp rIQ11** | **vpa 1st** | **vpa1st rIQ11** | **sym sear** | **sym sear rIQ11** | **mat reas** | **blk des** | **span B** | **spanB rIQ11** | **spanF** | **spanF rIQ11** | **dig**  **sym** | **dig sym rIQ11** | **ln seq** | **srt mean** | **Srt sd** |
| --- | --- | --- | --- | --- | --- | --- | --- | --- | --- | --- | --- | --- | --- | --- | --- | --- | --- | --- | --- | --- | --- | --- |
| **rs10492555** | 0,63 | 0,54 | 0,51 | 0,27 | 0,18 | 0,24 | 0,61 | 0,77 | 0,61 | 0,86 | 0,36 | 0,84 | 0,95 | 0,78 | 0,48 | 0,36 | 0,52 | 0,91 | 0,37 | 0,92 | 0,41 | 0,65 |
| **rs9315390** | 0,16 | 0,16 | 0,95 | 0,85 | 0,79 | **0,060** | 0,25 | 0,19 | 0,25 | 0,84 | 0,72 | 0,75 | 0,44 | 0,92 | 0,65 | 0,79 | 0,85 | 0,74 | 0,85 | **0,090** | 0,17 | 0,36 |
| **rs9315383** | 0,59 | 0,53 | 0,77 | 0,99 | 0,64 | **0,071** | **0,075** | **0,046** | **0,075** | 0,52 | 0,37 | 1,00 | 0,54 | 0,51 | 0,51 | 0,42 | 0,56 | 0,49 | 0,43 | 0,80 | 0,89 | 0,14 |
| **rs7334245** | **0,063** | **0,053** | 0,69 | 0,95 | 0,53 | 0,16 | 0,93 | 0,89 | 0,93 | 0,36 | 0,21 | 0,32 | **0,068** | 0,10 | **0,077** | 0,99 | 0,74 | 0,21 | 0,10 | 0,95 | 0,34 | 0,64 |
| **rs7989807** | 0,26 | 0,26 | 0,78 | 0,90 | 0,59 | 0,39 | 0,49 | 0,26 | 0,49 | 0,55 | 0,47 | 0,36 | 0,25 | 0,96 | 0,90 | 0,38 | 0,28 | 0,90 | 0,80 | 0,95 | 0,30 | 0,44 |
| **rs7323560** | 0,47 | 0,46 | 0,92 | 1,00 | 0,12 | 0,54 | **0,071** | 0,14 | **0,071** | 0,40 | 0,46 | 0,67 | 0,96 | 0,79 | 0,91 | 0,49 | 0,29 | 0,37 | 0,47 | 0,56 | **0,031** | **0,031** |
| **rs7989245** | 0,12 | **0,091** | 0,70 | 0,57 | **0,065** | 0,47 | 0,68 | 0,70 | 0,68 | 0,59 | 0,33 | 0,86 | 0,53 | 0,27 | 0,30 | 0,63 | 0,51 | 0,86 | 0,58 | 0,80 | 0,72 | 0,14 |
| **rs10507435** | 0,43 | 0,40 | 0,77 | 0,89 | 0,85 | 0,69 | 0,73 | 0,54 | 0,73 | 0,63 | 0,28 | 0,72 | **0,047** | 0,95 | 0,80 | 0,61 | 0,48 | 0,53 | 0,95 | 0,46 | 0,44 | 0,34 |
| **rs943220** | 0,11 | **0,076** | 0,88 | 0,96 | 0,82 | 0,91 | 0,84 | 0,90 | 0,84 | 0,97 | 0,69 | 0,21 | 0,17 | 0,79 | 0,98 | 0,96 | 0,99 | 0,63 | 0,94 | 0,99 | 0,63 | 0,98 |
| **rs4391923** | 0,84 | 0,80 | **0,0016** | **0,0023** | **0,042** | **0,100** | 0,24 | **0,072** | 0,24 | **0,021** | **0,048** | **0,049** | **0,011** | **0,011** | **0,032** | 0,27 | 0,32 | 0,16 | 0,34 | **0,082** | 0,71 | 0,88 |
| **rs2296645** | **0,034** | **0,028** | 0,46 | 0,46 | 0,56 | **0,039** | **0,030** | **0,087** | **0,030** | 0,13 | **0,040** | 0,43 | 0,64 | 0,92 | 0,79 | 0,85 | 0,90 | 0,14 | **0,034** | 0,33 | 0,55 | 0,19 |
| **rs1926467** | 0,46 | 0,52 | 0,94 | 0,80 | 0,40 | 0,20 | 0,13 | 0,27 | 0,13 | 0,20 | 0,57 | **0,014** | 0,39 | 0,34 | 0,48 | **0,011** | **0,0067** | 0,18 | 0,57 | **0,019** | 0,70 | 0,38 |
| **rs12430800** | 0,61 | 0,59 | 0,96 | 0,70 | 0,40 | 0,57 | 0,26 | 0,17 | 0,26 | 0,22 | 0,27 | 0,27 | 0,19 | 0,95 | 0,93 | 0,24 | **0,07** | 0,40 | 0,47 | 0,79 | 0,75 | 0,26 |
| **rs4591003** | 0,90 | 0,86 | 0,91 | 0,79 | 0,45 | 0,65 | 0,19 | 0,43 | 0,19 | 0,64 | 0,67 | **0,020** | **0,10** | 0,64 | 0,89 | **0,0073** | **0,0039** | 0,46 | 0,79 | 0,12 | 0,22 | 0,12 |
| **rs9545332** | 0,68 | 0,75 | 0,40 | 0,75 | **0,036** | 0,40 | 0,73 | 0,89 | 0,73 | 0,40 | 0,98 | 0,20 | **0,021** | 0,45 | 0,48 | 0,90 | 0,96 | **0,041** | 0,30 | **0,039** | 0,99 | 0,84 |
| **rs872060** | 0,36 | 0,28 | 0,72 | 0,73 | 0,30 | **0,083** | 0,46 | 0,51 | 0,46 | 0,60 | 0,77 | 0,82 | 0,28 | 0,95 | 0,71 | 0,97 | 0,96 | 0,93 | 0,69 | 0,35 | 0,89 | 0,51 |

### SOM Table 14: Haplotype trend regression analysis in the LBC1936

Two- and three- markers sliding windows haplotype trend regression was performed for the IQ variables. Age and sex were used as covariables. Association to IQ-70 was also tested using the IQ at age 11 as covariable.

|  | **IQ-11** |  |  | **IQ-70** |  |  |  |  |  |
| --- | --- | --- | --- | --- | --- | --- | --- | --- | --- |
| **Variable** | **LR** | **HTR2** | **HTR3** | **LR** | **LR r11** | **HTR2** | **HTR2 rIQ11** | **HTR3** | **HTR3 rIQ11** |
| **rs10492555** | **0.0568** | 0.3878 | 0.4725 | 0.8999 | 0.3764 | 0.9387 | 0.9067 | 0.9825 | 0.5610 |
| **rs9315390** | 0.5278 | 0.5357 | 0.8045 | 0.6796 | 0.7433 | 0.9623 | 0.7348 | 0.9630 | 0.6319 |
| **rs9315383** | 0.6715 | 0.9585 | 0.9144 | 0.9184 | 0.9759 | 0.8686 | 0.6559 | 0.9194 | 0.8275 |
| **rs7334245** | 0.8090 | 0.5106 | 0.5552 | 0.4695 | 0.3959 | 0.5293 | 0.7964 | 0.5743 | 0.7710 |
| **rs7989807** | 0.9293 | 0.9515 | 0.9538 | 0.5740 | 0.5138 | 0.8348 | 0.6824 | 0.9464 | 0.6512 |
| **rs7323560** | 0.7124 | 0.6453 | 0.9744 | 0.5858 | 0.6393 | 0.9294 | 0.7551 | 0.9541 | 0.9149 |
| **rs7989245** | 0.8287 | 0.9644 | 0.1065 | 0.8147 | 0.3737 | 0.8705 | 0.8845 | 0.4254 | 0.9584 |
| **rs10507435** | 0.6472 | 0.4866 | 0.3956 | 0.4841 | 0.8581 | 0.4683 | 0.8772 | **0.0092** | **0.0941** |
| **rs943220** | 0.3827 | 0.2522 | 0.5883 | 0.3044 | 0.5531 | **0.0026** | **0.0282** | **0.0423** | **0.0260** |
| **rs4391923** | 0.1489 | 0.3799 | 0.1640 | **0.0101** | **0.0262** | **0.0917** | **0.0420** | **0.0787** | **0.0566** |
| **rs2296645** | 0.3699 | **0.0712** | **0.0445** | 0.5969 | **0.0794** | **0.0639** | 0.1113 | 0.1962 | 0.1786 |
| **rs1926467** | **0.0225** | **0.0189** | **0.0733** | 0.1251 | 0.5624 | 0.3092 | 0.4731 | 0.6017 | 0.9044 |
| **rs12430800** | 0.5540 | **0.0489** | 0.1066 | 0.6747 | 0.8774 | 0.4063 | 0.7193 | 0.5867 | 0.8373 |
| **rs4591003** | **0.0157** | 0.1031 | **0.0848** | 0.2850 | 0.7582 | 0.6632 | 0.4934 | 0.7646 | 0.8386 |
| **rs9545332** | **0.0231** | **0.0516** |  | 0.2125 | 0.7501 | 0.6214 | 0.7163 |  |  |
| **rs872060** | 0.8631 |  |  | 0.9890 | 0.7737 |  |  |  |  |

## Results from sequencing of human DCLK1 intron 5.

The total intron 5 (chr13: 35342944 – 35326594, total 16 350 bp) was sequenced in 23 individuals from the NCNG sample. Because of high repeat content the following regions could not be sequenced:

1. 35,340,183 – 35,339,984: contains a complex and polymorphic CTTTTCT repeat (confirmed by PCR).
2. 35,337,063 – 35,336,194: contains a polymorphic insertion/deletion of an ALU-Y repeat.
3. 35,334,205 – 35,33,998: long stretches of Ts

Following table: list of SNPs observed in the panel of 23 individuals (46 chromosomes) sequenced. When already described, the dbSNP reference ID is given. All other newly observed SNPs have been submitted to dbSNP and will appear in dbSNP 130 under the identifier “MARTENS”. Additional dbSNPs for the region which were not observed in our sample are not listed.

| **dbSNP id** | **In house ID** | **chromEnd** | **observed** | **Within regulatory region?** |
| --- | --- | --- | --- | --- |
| rs1926452 |  | 35342937 | A/G |  |
| rs17786591 |  | 35342633 | A/C |  |
| rs17786544 |  | 35342147 | A/G |  |
| submitted | 1.d | 35342061 | A/G |  |
| submitted | 2.a ins/del | 35342746 | INS/DEL |  |
| rs11838747 |  | 35341674 | G/T |  |
| rs11147590 |  | 35341100 | C/G |  |
| rs12428685 |  | 35341088 | C/T |  |
| rs12427644 |  | 35341000 | C/T |  |
| submitted | 4.a | 35340246 | T/C | cis 1 |
| rs9593516 |  | 35340034 | A/G |  |
| rs9565612 |  | 35340015 | A/G |  |
| rs12100412 |  | 35339752 | G/T |  |
| rs9601513 |  | 35339438 | A/C |  |
| submitted | 5.b | 35339397 | A/G |  |
| submitted | 5.c | 35339342 | A/G |  |
| rs12428100 |  | 35339156 | C/T | cis 2 |
| rs12428086 |  | 35339099 | C/T | cis 2 |
| rs12431039 |  | 35339013 | C/T | cis 2 |
| rs10507435 |  | 35338996 | A/G | cis 2 |
| rs10507434 |  | 35338404 | C/G |  |
| rs17053012 |  | 35337817 | C/T |  |
| submitted | 7.a | 35338049 | A/G |  |
| rs4633579 |  | 35337435 | G/T |  |
| submitted | 7.b | 35337397 | A/T |  |
| rs943220 |  | 35337252 | C/T |  |
| submitted | 7.c | 35337115 | A/C |  |
| rs6145001 |  | 35336722 | Large deletion | cis 3 |
| submitted | 9.a | 35336192 | A/G |  |
| rs17181655 |  | 35336130 | C/T |  |
| rs17181641 |  | 35335947 | G/T |  |
| rs17181606 |  | 35335744 | C/G |  |
| rs12427459 |  | 35335371 | C/T |  |
| rs12427442 |  | 35335182 | C/G |  |
| submitted | 10.a | 35335071 | A/C |  |
| submitted | 10.b | 35335040 | C/T |  |
| rs17181551 |  | 35334924 | C/T |  |
| submitted | 11.a | 35333956 | C/G |  |
| rs13378928 |  | 35333568 | C/T |  |
| submitted | 12.a | 35333462 | C/T |  |
| submitted | 13.a | 35332667 | C/T |  |
| rs2148417 |  | 35331862 | A/G |  |
| rs4943344 |  | 35331036 | C/T |  |
| rs4941821 |  | 35330371 | C/T |  |
| submitted | 15.a | 35330139 | C/T |  |
| rs9574747 |  | 35329698 | C/T |  |
| rs4391923 |  | 35328856 | A/G |  |
| submitted | 17.b | 35328406 | A/G |  |

We observed 4 SNPs within the cis 2 region and 2 additional SNP close to the region, which could potentially alter the transcription factors binding efficiency. In order to clone the cis 2 region haplotypes from genomic DNA, we screened the sequenced individuals for homozygotes for the region. Thirteen homozygous individuals were identified, which presented the 3 following haplotypes:

| SNP |  |  |  | Hap 1a | Hap 1b | Hap 2 |
| --- | --- | --- | --- | --- | --- | --- |
| 5.b | 35339397 | A/G |  | G | A | G |
| 5.c | 35339342 | A/G |  | A | G | A |
| rs12428100 | 35339156 | C/T | cis 2 | T | T | C |
| rs12428086 | 35339099 | C/T | cis 2 | T | T | C |
| Rs12431039 | 35339013 | C/T | cis 2 | C | C | T |
| Rs10507435 | 35338996 | A/G | cis 2 | A | A | G |
|  | | | |  |  |  |
| Number of observations in homozygosity | | | | 5 | 2 | 6 |

Hap 1a and Hap 1b differed only for SNPs 5.b and 5.c which are outside of the predicted cis 2 region. Hap 2 and Hap 1a/b differed for their haplotype in the cis 2 region.

## Prediction of promoter and regulatory regions of human DCLK1 intron 5.

| 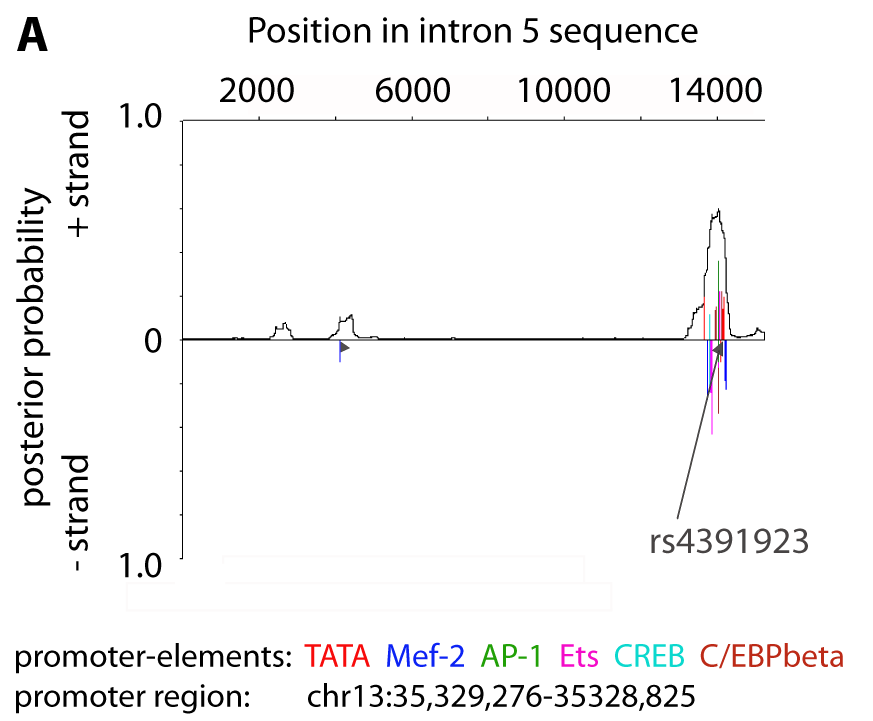 | 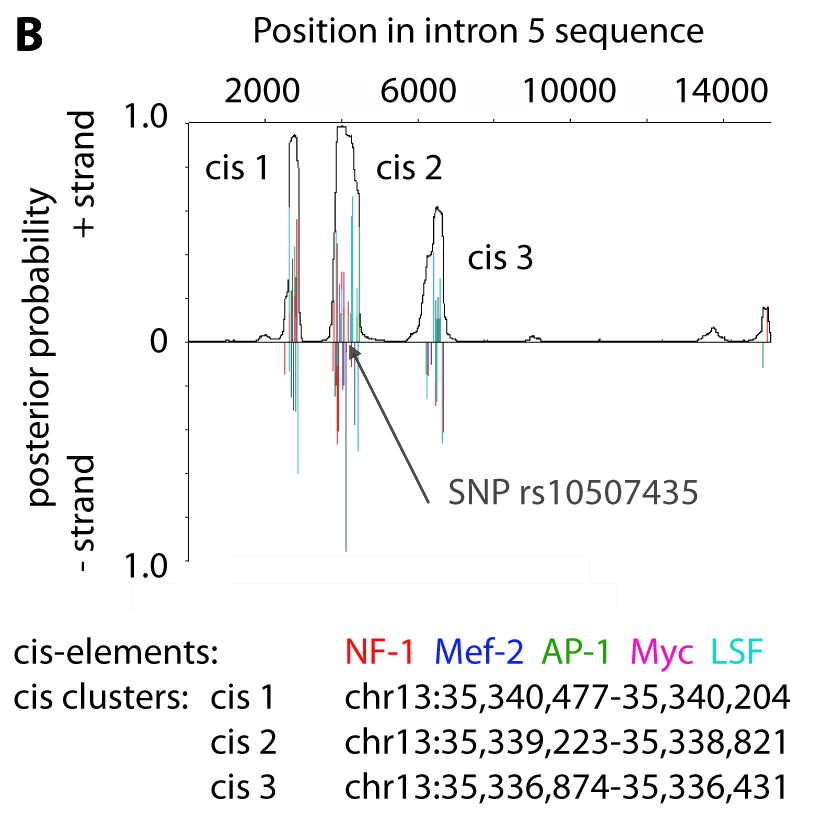 |
| --- | --- |

*In silico* prediction of promoter and regulatory regions in human *DCLK1* intron 5. (A) The intron 5 sequence contained 24 possible TATA-boxes (probability scores = 0.85-1.0), as identified by the neuronal promoter prediction program (http://www.fruitfly.org/seq_tools/ promoter.html). Only one TATA-box was found to cluster with several common promoter elements, using the cis-element cluster characterization (Cister)-program (http://zlab.bu.edu/%7Emfrith/cister.shtml). The predicted promoter includes the marker m5.3 (rs4391923) located three bp upstream of the transcriptional start site. (B) Cluster probability of TFs in the region surrounding marker m5.1 (rs10507435).

## Expression of human CARP mRNA in cultured SH-SY5Y cells.

*
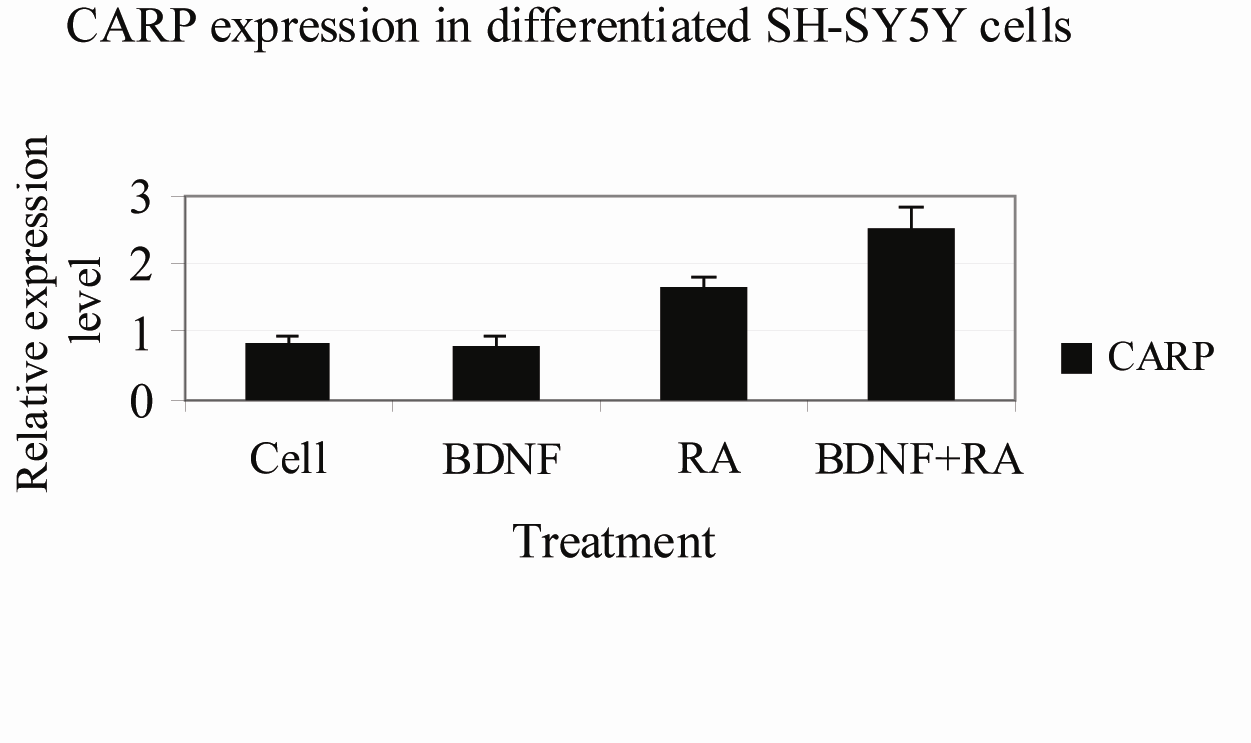
*

Neuronal differentiation of SH-SH5Y was performed as described (3), and shown to induce the expression of endogenous human *CARP* mRNA. The highest *CARP* expression level was observed in response to BDNF and retinoic acid (RA) (*CARP* mRNA: RA, fold change=1.64±0.15; BDNF/RA, fold change =2.51±0.32; n = 4). The effect of neuronal differentiation on luciferase reporters with predicted *DCLK1* intron 5 regulatory elements (cis2 and C/Tprom) were analyzed in response to BDNF+RA.

# References

1. Weschler D (1998) WMS-IIIUK administration and scoring manual. San Antonio: Psychological Corporation.

2. Weschler D (1987) Weschler Memory Scale - Revised. San Antonio: The Psychological Corporation.
